# Diet and specific microbial exposure trigger features of environmental enteropathy in a novel murine model

**DOI:** 10.1038/ncomms8806

**Published:** 2015-08-04

**Authors:** Eric M. Brown, Marta Wlodarska, Benjamin P. Willing, Pascale Vonaesch, Jun Han, Lisa A. Reynolds, Marie-Claire Arrieta, Marco Uhrig, Roland Scholz, Oswaldo Partida, Christoph H. Borchers, Philippe J. Sansonetti, B. Brett Finlay

**Affiliations:** 1 Department of Microbiology and Immunology, University of British Columbia, Vancouver, British Columbia, Canada; 2 Michael Smith Laboratories, University of British Columbia, Vancouver, British Columbia, Canada V6T 1Z4; 3 Department of Agricultural, Food and Nutritional Science, University of Alberta, Edmonton, Alberta, Canada T6G 2P5; 4 Molecular Microbial Pathogenesis Unit, Institut Pasteur, Paris 75724, France; 5 The UVic-Genome British Columbia Proteomics Centre, University of Victoria, Victoria, British Columbia, Canada V8Z 7X8; 6 Department of Biochemistry and Microbiology, University of Victoria, Victoria, British Columbia, Canada V8P 5C2; 7 Department of Biochemistry and Molecular Biology, University of British Columbia, Vancouver, British Columbia, Canada V6T 1Z4

## Abstract

Environmental enteropathy (EE) is a subclinical chronic inflammatory disease of the small intestine and has a profound impact on the persistence of childhood malnutrition worldwide. However, the aetiology of the disease remains unknown and no animal model exists to date, the creation of which would aid in understanding this complex disease. Here we demonstrate that early-life consumption of a moderately malnourished diet, in combination with iterative oral exposure to commensal Bacteroidales species and *Escherichia coli*, remodels the murine small intestine to resemble features of EE observed in humans. We further report the profound changes that malnutrition imparts on the small intestinal microbiota, metabolite and intraepithelial lymphocyte composition, along with the susceptibility to enteric infection. Our findings provide evidence indicating that both diet and microbes combine to contribute to the aetiology of EE, and describe a novel murine model that can be used to elucidate the mechanisms behind this understudied disease.

## Abstract (web summary)

Environmental enteropathy is a disorder of the small intestine that contributes to the persistence of childhood malnutrition worldwide. Here, Brown *et al*. show in mice that early-life malnourishment, in combination with exposure to commensal bacteria, remodels the small intestine to resemble features of the disease.

## Graphical abstract

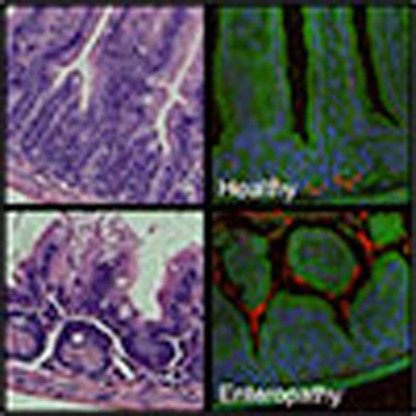

Malnutrition is a significant worldwide health issue, causing one-fifth of all deaths in children <5 years of age^1^. Early-life malnutrition results in childhood growth stunting and decreased cognitive development^2^,^3^, along with significant changes in intestinal microbiota composition^4^. During the first 2 years of life in malnourished children, there is an immature development of the intestinal microbiota compared with healthy, case-controlled children^5^, and a comprehensive study across multiple geographical regions concluded that children consistently harbour a significantly more diverse early-life microbiota composition in low socioeconomic regions of the world^6^. Clinical trials have shown that therapeutic food interventions are not sufficient to aid in the development of a healthy early-life intestinal microbiota and fail to fully restore health in malnourished children^5^,^7^,^8^. However, antibiotic treatment preceding the nutritional intervention leads to an improvement of the health defects cause by malnutrition, indicating the importance of understanding the microbial-driven disease pathologies and underlying defects in intestinal function contributing to malnutrition^9^.

A poorly understood disorder of small intestine called environmental enteropathy (EE), also referred to as tropical enteropathy or environmental enteric dysfunction, is now widely recognized to be a major contributor to childhood malnutrition^10^,^11^,^12^, and is thought to be the reason why current therapeutic interventions in malnourished children are efficacious less than one-third of the time^13^. This disease impacts the function of the small intestine, and features include chronic intestinal inflammation, villous blunting and increased intestinal permeability, ultimately resulting in diminished nutrient uptake, adversely affecting growth and development^14^,^15^. As a subclinical disorder, EE presents without diarrhoea or presence of any known infectious aetiology, unlike tropical sprue^10^, and is mainly observed in regions of the world with poor sanitation and hygiene, suggesting that microbial exposure or the microbiota play a role in its development^16^. Until recently, studies incorrectly assumed that diarrhoea was the primary driver of early-life growth stunting, whereas now it is recognized that many of the sequelae of malnutrition happen independently of diarrhoea^17^. The aetiology of EE has not been experimentally demonstrated, but one hypothesis in the literature is that the increased ingestion of faecal-associated bacteria early in life (due to poor sanitation and hygiene) can lead to a pathological shift in microbe–host interactions in the small intestine and chronic inflammation exacerbating malnutrition^15^,^18^,^19^. This hypothesis would also explain the benefit of antibiotic treatment before therapeutic food interventions for malnourished children. Unfortunately, EE is largely understudied owing to the difficulty in obtaining samples directly from the small intestine in humans, which requires invasive biopsies deemed unethical because of the subclinical nature of EE. Thus, insight into the connection between metabolism, microbiota and immunity in the malnourished intestine is limited. Furthermore, many human studies take only faecal samples to assess the microbiota composition in malnutrition and EE cases, yet this does not capture the small intestinal bacterial community, an important consideration, since the faecal microbiota composition is not representative of those microbes colonizing the small intestine^20^ where pathology of this disease occurs. Thus, there is a major unmet need for a preclinical animal model of EE, and these models will be crucial to understand the pathogenesis of EE, its impact on malnutrition and to develop therapies^16^.

Here we describe the first animal model of EE and we demonstrate that both diet and oral microbial exposure to specific bacteria are required for the induction of EE in a mammalian host. Utilizing this model, we were able to achieve for the first time, an in-depth characterization of the impact of malnutrition on the microbiota, metabolism and immune system in the mammalian small intestine, along with assessing the impact of enteric infection. These studies provide a preclinical model to test therapeutics and will enable a greater understanding of the pathophysiological nature of EE.

## Results

### Impact of malnutrition on growth and gut barrier function

**Figure 1:**
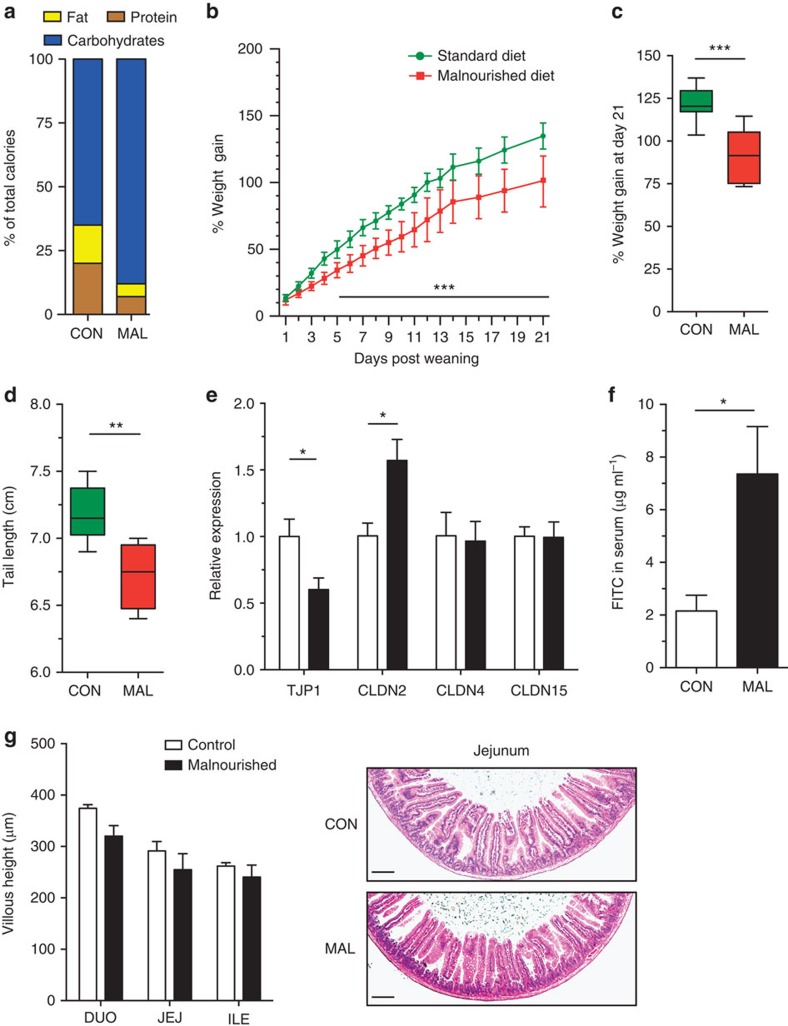
Assessment of the growth rate and intestinal barrier function in C57BL/6 mice fed a malnourished or isocaloric control diet. (**a**) A schematic of the components of each diet, expressed as a per cent of total calories. (**b**) Mice were given each diet post weaning, and daily weight change was assessed daily over a period of 21 days. Data are representative of the three independent experiments, *n*=8 mice per group. Data points represent the mean and error bars indicate the s.d. (***P<0.001, repeated measures analysis of variance). After 3 weeks of being fed each diet, the total amount of (**c**) weight gained and (**d**) final tail lengths were calculated. In the box and whisker plots shown, the middle bar represents the mean, the bottom and top of the box are the first and third quartiles, respectively, and whiskers indicate the range of the data. Data are representative of three independent experiments, *n*=8 mice per group (Student's *t*-test **P<0.01). (**e**) The jejunal mRNA expression of *TJP1*, *CLDN2*, *CLDN4* and *CLDN15* are expressed as fold change relative to the control-fed mice. Bars indicate the mean with s.e.m., and are representative of two independent experiments, eight mice per group (*P<0.05, Student's *t*-test). (**f**) Concentration of FITC in the serum was assessed 4 h post administration orally, after mice were fed each diet for 3 weeks. Bars indicate the mean with s.e.m., and are representative of two independent experiments, *n*=8 per group (*P<0.05, Student's *t*-test). (**g**) The average measured villous height in the duodenum (DUO), jejunum (JEJ) and ileum (ILE) with representative H&E-stained images of jejunum sections from mice exposed to each diet. Scale bar, 100 μm (in length). Bar graph indicates the mean with s.e.m., and is representative of three independent experiments, eight mice per group. CON, control; MAL, malnourished.

We first sought to characterize the impact of a moderately malnourished diet on the growth rate, intestinal architecture and barrier function of the small intestine in young mice. Post weaning, mice were randomized into receiving either a malnourished diet (7% protein and 5% fat) or isocaloric control diet (20% protein and 15% fat; Fig. 1a and Supplementary Table 1). Both groups consumed equivalent weights per day of the chow (Supplementary Fig. 1a). Mice fed the malnourished diet gained significantly less weight over time in comparison with mice fed the isocaloric control diet (Fig. 1b). Growth stunting of the malnourished mice was evident as they gained an average of 30% less weight after 21 days (Fig. 1c). At this time point, malnourished mice also had considerably shorter tails, a surrogate marker for length of the mouse (Fig. 1d). There was a decreased expression of insulin-like growth factor 1, which encodes a growth factor hormone, and angiotensin-converting enzyme 2, which is necessary for protein uptake^21^, in the jejunum of malnourished mice (Supplementary Fig. 1b,c). Next, the barrier function of the jejunum was assessed by analysing mRNA expression levels of tight-junction protein-1 (*TJP1*), claudin-2 (*CLDN2*), claudin-4 (*CLDN4*) and claudin-15 (*CLDN15*). *TJP1*, the product of which is ZO-1, a major component of tight-junction assembly and function^22^, was expressed at lower levels in malnourished mice (Fig. 1e). In contrast, the expression of *CLDN2* was upregulated in the jejunum of malnourished mice, which is also indicative of increased permeability based on previous reports^23^ (Fig. 1e). However, the expression of other claudin transcripts (*CLDN4/15*) was similar between the groups of mice (Fig. 1e). To measure whether these gene expression changes also had a functional impact on barrier function, mice were fed fluorescein isothiocyanate (FITC)-labelled dextran and subsequent FITC levels were measured in the serum. There was a 3.5-fold increase in the serum concentration of FITC in malnourished mice compared with controls, indicating an increase in intestinal permeability (Fig. 1f). Barrier dysfunction did not significantly impact total secretory immunoglobulin A (IgA) levels in the jejunum, an important antibody for host epithelial defence (Supplementary Fig. 1d). In addition, sections of the small intestine (duodenum, jejunum and ileum) were visualized microscopically and no significant signs of inflammation, goblet cell depletion, histopathology or villous blunting were observed in the mice fed the malnourished diet (Fig. 1g and Supplementary Fig. 1e). These data suggest that the malnourished diet led to a moderate growth stunting and increased intestinal permeability relative to the isocaloric control diet independent of small intestinal histopathology.

### Malnutrition induces an altered small intestinal microbiota

**Figure 2:**
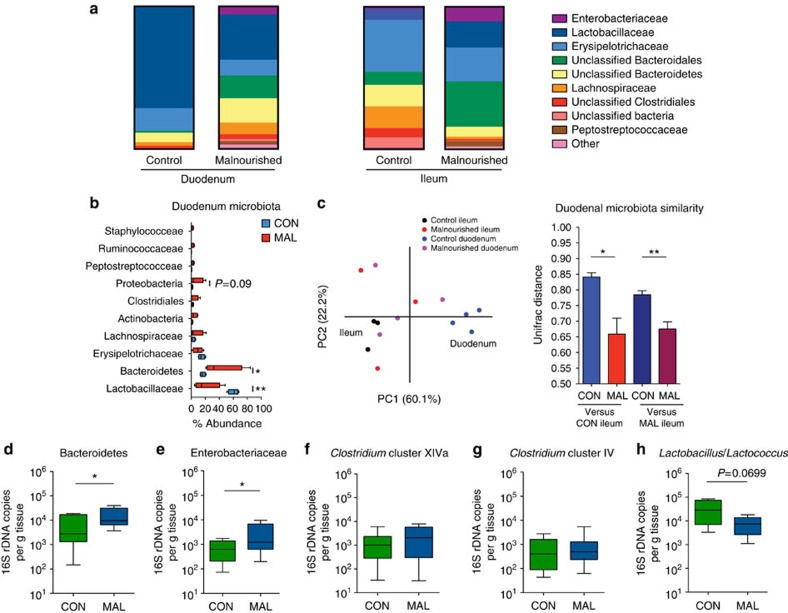
Relative abundance of the small intestinal microbiota in malnourished and control mice. (**a**) A chart summarizing the pooled per cent abundance of the duodenal (*n*=4) and ileal (*n*=3) microbiota by family classification using the 16S rRNA gene. (**b**) A PCA plot of the microbial communities in the duodenum (*n*=4) and ileum (*n*=3) of mice on each diet. Communities were plotted based on a measure of UniFrac distance between the communities. This UniFrac distance measure was used to quantify the similarity between the microbiota in the duodenum of malnourished and control mice to the control ileum (left two bars) and the malnourished ileum (right two bars). A lower value indicates greater community similarity. Bars indicate the mean with s.e.m. (**c**) OTUs at the phylum and family level of taxonomy were plotted on a box and whisker graph to show changes in per cent abundance relative to the total number of OTUs in the duodenum of malnourished and control mice. OTUs from the Lactobacillaceae (**P<0.01), Bacteroidetes (*P<0.05) and Proteobacteria (*P*=0.09) were the three most significantly changed taxa between the mice (Mann–Whitney *U*-test). (**d**–**h**) Real-time qPCR analysis of (**d**) mouse intestinal Bacteroidetes-specific 16S rDNA, (**e**) Enterobacteriaceae-specific 16S rDNA, (**f**) *Clostridium* cluster XIVa 16S rDNA, (**g**) *Clostridium* cluster IV 16S rDNA and (**h**) *Lactobacillus/Lactococcus* in each gram of duodenal tissue. Analysis was performed on DNA extracted from duodenal tissue of malnourished and control mice. Data are pooled from 3 independent experiments, 12 mice per group, and bars indicate the mean with s.e.m. (*P<0.05, Student's *t*-test). CON, control; MAL, malnourished.

To test whether malnutrition alters the small intestinal microbiota in mice, we performed 454 pyrosequencing of the 16S rRNA genes from the microbiota in the duodenum and ileum of the mice 3 weeks after being fed the malnourished or isocaloric control diet (Supplementary Data 1). We found marked shifts in the bacterial species composition of both the ileum and duodenum in the malnourished mice, compared with those on the control diet (Fig. 2a). The ileum of malnourished mice showed both an expansion of species from the Bacteroidetes and Proteobacteria phyla, and decrease in the Lachnospiraceae and Clostridiales families compared with control mice (Fig. 2a and Supplementary Fig. 2a). The duodenum of malnourished mice also had an abnormal and significant expansion in the proportion of bacteria belonging to the phyla Bacteroidetes and Proteobacteria (largely the Enterobacteriaceae family; Fig. 2a,b and Supplementary Fig. 2a,b), which are normally found at high abundance in the lower intestinal tract nearer the colon in C57BL/6 mice^24^. Normal residents of the duodenum, the Gram-positive bacteria from Lactobacillaceae and Erysipelotrichaceae families were decreased in abundance in the malnourished duodenum with a striking 45% reduction in the abundance of Lactobacillaceae (Fig. 2b). This decrease coincided with an increase in abundance of a more diverse set of operational taxonomic units (OTUs) from the Bacteroidales*, Prevotella*, Lachnospiraceae, Clostridiales, *Pseudomonas Escherichia*, Peptostreptococcaceae and Ruminococcaceae groups (Supplementary Table 2). As visualized on the principal component analysis (PCA) plot, the dietary changes cause the microbial community of the duodenum to have greater similarity with the ileum microbial community, and this can be quantified by assessing the UniFrac distance, a measure of community similarity (Fig. 2c). Using this measure, we found that the resident microbiota in the malnourished duodenum was significantly more similar (*P*<0.01, Student's *t*-test) to the ileal community than the community in the control duodenum samples (Fig. 2c). There was also a marked increase in bacterial diversity of the duodenum in malnourished mice compared with those in the control diet, as seen in the phylogenetic distribution at the genus level and quantified using the inverse Simpson's diversity index (Supplementary Fig. 2b,c). Furthermore, quantitative PCR analysis of 16S copies in the duodenal microbiota verified that the family-level microbial differences observed in the pyrosequencing data were consistent across multiple experiments (Fig. 2d–h). Overall, these data suggest that the malnourished diet remodelled the bacterial community in the duodenum to more closely resemble the ileum, where Gram-negative species (Bacteroidetes and Proteobacteria) were more abundant, displacing the indigenous Gram-positive Firmicutes, such as Lactobacillaceae (Supplementary Fig. 2d).

### Impact of malnutrition on small intestinal metabolome

**Figure 3:**
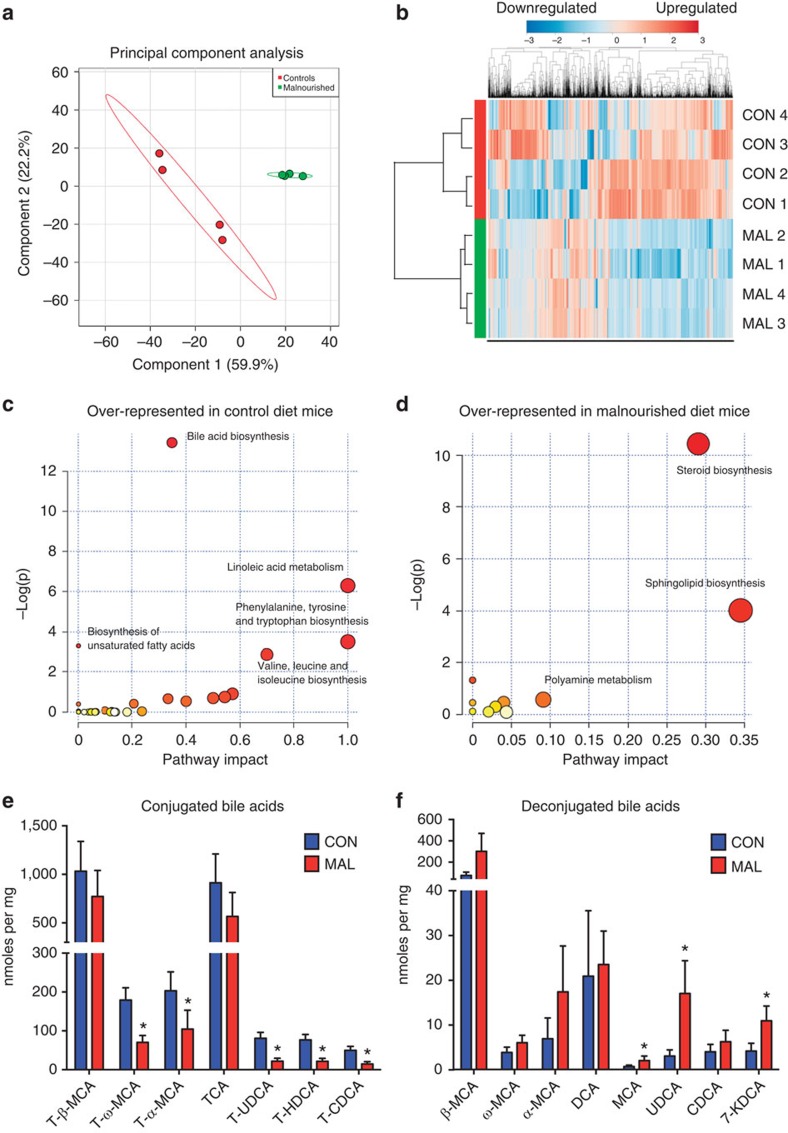
Untargeted and targeted metabolomics of the small intestinal metabolome. (**a**) A PCA plot showing separation of metabolomic data as detected by the positive ion channel from malnourished mice (green) and control mice (red). (**b**) A heat map of the relative abundance of all metabolites identified from the small intestinal metabolome as detected by the positive ion channel from malnourished and control mice (*n*=4). The malnourished (MAL) and control (CON) samples clustered together in the dendogram based on cluster analysis by the Ward method, with a Pearson distance measure. The heat map scale is a log_2_ base, from the range of −3 (blue) to +3 (red). (**c**,**d**) Plots showing over-represented pathways using Metaboanalyst 2.5 software in (**c**) control mice and (**d**) malnourished mice, as identified by the Kyoto Encyclopedia of Genes and Genomes database. The plot is graphed based on the log(*P* value; Welch's *t*-test; *y* axis) and per cent of pathway impacted (*x* axis). The size of the circles represents the number of metabolites identified to be part of the pathway. (**e**) Tauro-conjugated bile acid and (**f**) unconjugated bile acid concentrations in the small intestine of malnourished (*n*=3) and control (*n*=4) as determined by targeted metabolomics. Bars represent the mean±s.e.m., (*P<0.05, Mann–Whitney *U*-test). CA, cholic acid; CDCA, chenodeoxycholic acid; HDCA, hyodeoxycholic acid; KDCA, ketodeoxycholic acid; MCA, muricholic acid; T, tauro conjugation; UDCA, ursodeoxycholic acid.

As malnutrition leads to drastic shifts in the intestinal microbiota, we hypothesized that this would also impact the metabolic environment of the host small intestine. After 3 weeks of treatment with either diet and concurrent with our microbial analysis, contents of the small intestine in malnourished and control diet mice were assessed for abundance changes of the metabolites using untargeted metabolomic analysis by ultrahigh-performance liquid chromatography–Fourier transform (FT) mass spectrometry (UPLC–FTMS). The method detected and relatively quantified over 3,500 unique metabolite features, and ∼420 showed differential abundances between malnourished and control mice (*P*<0.05; >2-fold change). Overall, the metabolite profile of the malnourished small intestine substantially shifted from the profile of control mice, as shown by unsupervised PCA (Fig. 3a). When all metabolites detected in the positive ion mode were visualized on a heat map, malnourished and control groups clustered together in the dendogram indicating that the diet shifted the metabolome to an alternate state (Fig. 3b). Each of these observations held true for metabolites detected on the negative ion channel (Supplementary Fig. 3a,b).

Using the metabolite matches based on mass to charge ratio (*m/z*) values found with the Kyoto Encyclopedia of Genes and Genomes database, we determined specific pathways that were enriched by the malnourished or control diet. Metabolites in the bile acid biosynthesis pathway (37% of pathway impacted; Fisher's exact test *P*<0.001), linoleic acid metabolism and amino-acid biosynthesis were all significantly over-represented in the control diet (Fig. 3c). The changes in the amino-acid biosynthesis pathways were expected in the control mice, given the higher protein content of their diet, supporting the accuracy of our method and analysis. The most striking over-representation in malnourished mice was the greater abundance of metabolites in the steroid biosynthesis pathway (28% of pathway impacted; *P*<0.001) (Fig. 3d). More specifically, many of these steroid metabolites represented those from the vitamin D biosynthesis pathway. Random forest analysis was implemented to identify discriminatory features between the two diets, and it ranked a vitamin D metabolite as the most significant feature between the samples from the malnourished mice and controls (Supplementary Table 3). Of note, the vitamin D pathway is known to impact many aspects of intestinal homeostasis, including gut inflammation^25^ and bile acid production^26^. We confirmed these metabolome changes with a targeted metabolomics approach to quantify the concentrations of bile acids, vitamins and microbial-produced short-chain fatty acids (SCFAs) in the small intestine. Bile acid-targeted metabolomics revealed a number of significant changes in the bile acid pool between the malnourished and control small intestine, supporting the pathway analysis data from the untargeted metabolomics screen (Fig. 3e,f). In the malnourished small intestine, there was a shift towards lower concentrations of tauro-conjugated bile acids, and, conversely, higher levels of unconjugated bile acids, with the ratio between the two being significantly altered (Supplementary Fig. 4a–c). Each tauro-conjugated bile acid detected in this analysis was lower in the malnourished small intestine, with the concentrations of tauro-ω-muricholic acid, tauro-α-muricholic acid, tauroursodeoxycholic acid, taurohyodeoxycholic acid and taurochenodeoxycholic acid reaching statistical significance (Mann–Whitney *U*-test; Fig. 3e). In the unconjugated pool, the concentrations of muricholic acid, ursodeoxycholic acid and 7-ketodeoxycholic acid were all significantly higher in the malnourished small intestine (Mann–Whitney *U*-test; Fig. 3f). Results from the vitamin-targeted metabolomics analysis showed that the malnourished diet induced large shifts in the luminal concentrations of many vitamins in the small intestine (Supplementary Fig. 5), which are not due to altered concentrations of vitamins in the diets as they are identical (Supplementary Table 1). Malnutrition also induced significant reductions in vitamin A metabolites (retinol and retinal), vitamin B1, vitamin B6, vitamin B12 and vitamin E (α-tocopherol), and elevated concentrations of vitamin D3 and vitamin B7 (Supplementary Fig. 5). No significant changes in SCFAs were observed, although there was a general trend towards greater total concentrations of each in the malnourished small intestine (Supplementary Fig. 6). These data clearly show that a malnourished diet exerts a strong, measurable impact on both the microbial and metabolite content in the small intestine, mainly in the biosynthesis or bioavailability of steroids, vitamins and bile acids, and may be contributing to or a result of changes in bacterial composition.

### A Bacteroidales and *E. coli* cocktail replicates features of EE

**Figure 4:**
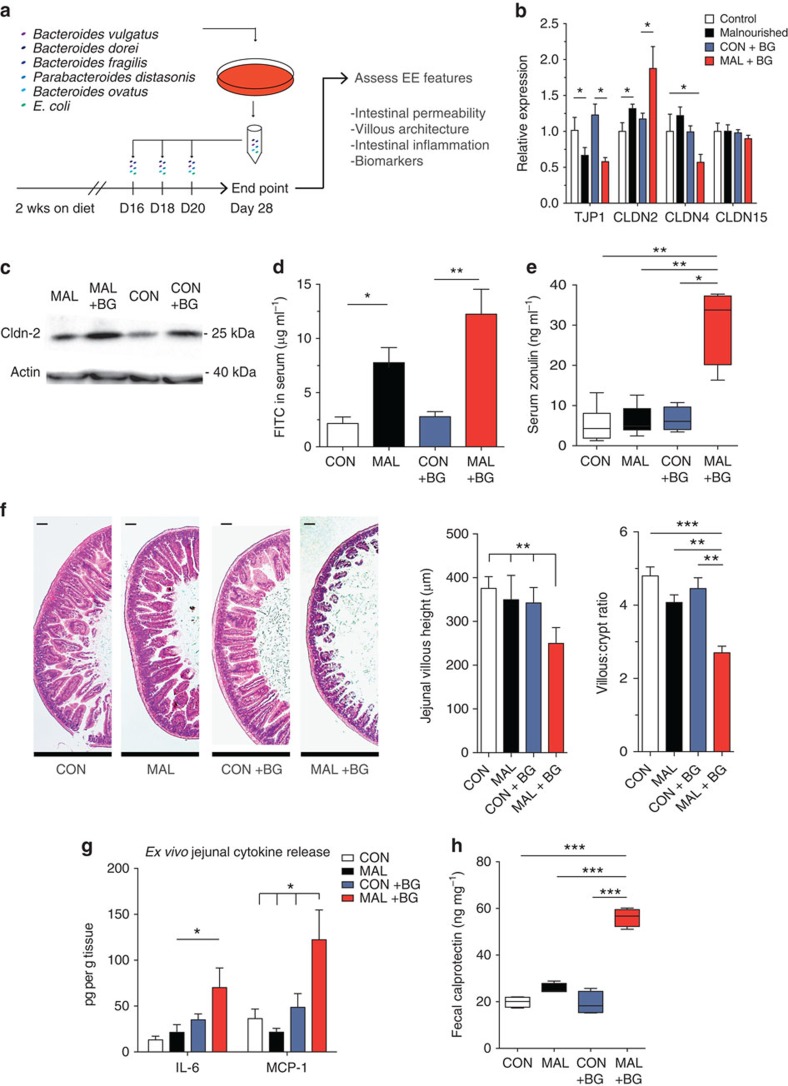
Characterizing the impact of Bacteroidales–*E. coli* oral exposure on small intestinal histopathology, inflammation and intestinal permeability in malnourished and control mice. (**a**) A schematic of the experimental design used to administer the Bacteroidales*–E. coli* cocktail. (**b**) The jejunal mRNA levels of *TJP1*, *CLDN2*, *CLDN4* and *CLDN15* were determined on unexposed mice on each diet (white and black bars), and BG-exposed mice on each diet (blue and red bars). The values are expressed as fold change relative to the control, unexposed mice. Representative of three independent experiments, *n*=5 per group. (**c**) Levels of CLDN2 protein blotted from extracted protein from jejunal IECs relative to the actin control. Blot is representative of three samples and two independent experiments (Supplementary Fig. 14). (**d**) Four hours after FITC–dextran administration, concentration of FITC in the serum in malnourished and control mice (*n*=8), and BG-exposed malnourished and control mice (*n*=8). Representative of two independent experiments. (**e**) Box and whisker plot of the concentration of zonulin in the sera of malnourished and control mice (*n*=8) and BG-exposed malnourished and control mice (*n*=8). Data are pooled from two independent experiments. (**f**) Histological assessment of H&E-stained jejunal tissues from BG-exposed and unexposed malnourished and control mice. Scale bars, 100 μm (in length). Images and graphs are representative of three independent experiments, five mice per group. (**g**) Concentrations of IL-6 and MCP-1 released in cultured jejunal tissue sections from BG-exposed and unexposed malnourished and control mice as measured by ELISA. Data are representative of two independent experiments, five mice per group. (**h**) Box and whisker plot of the concentration of faecal calprotectin in BG-exposed or unexposed malnourished mice (*n*=4 per group). All data in standard bar graphs are presented as the means with s.e.m. Box and whisker plots have a middle band that represents the mean, the bottom and top of box are the first and third quartiles, respectively, and the whiskers indicate the range of the data. Statistical analysis was performed using the one-way analysis of variance with *post hoc* Tukey's test (*P<0.05, **P<0.01, ***P<0.001). CON, control; MAL, malnourished; Wks, weeks.

Given the malnourished-induced shifts in the small intestinal microbiota and metabolome, we postulated that oral exposure to only specific species of bacteria would be necessary to induce EE features. We utilized the small intestinal microbial data to rationally select taxonomically grouped mixtures of species with a propensity to colonize the small intestine more readily in malnourished mice, and, subsequently, we orally exposed mice on both diets to these cocktails of cultured bacterial species (Supplementary Fig. 7 and Supplementary Table 4). In this model, young mice were randomized onto either the control or malnourished diet for 2 weeks, then gavaged every 2 days with each bacterial cocktail for an additional 6 days and finally the characteristic pathological features of EE were assessed 1 week after the last gavage (Fig. 4a). Alternatively, to measure growth rates, malnourished mice were gavaged every 3 days post weaning for a total of 2 weeks, and EE features were assessed 1 week later (Supplementary Fig. 8). Remarkably, we found that in combination with the malnourished diet, the oral exposure to defined mixture of Bacteroidales and *E. coli* (BG) was able to trigger villous blunting and exacerbate the effect of the malnourished diet on growth stunting, intestinal permeability and inflammation resulting in intestinal disease, which is strikingly similar to features of human EE (Fig. 4b–h and Supplementary Fig. 8a–e). Importantly, mice given a standard diet and exposed to BG did not show any pathological consequences and maintained normal growth compared with untreated, control mice (Supplementary Fig. 8a,b).

Malnourished mice exposed to BG showed increased growth stunting (35% less weight) as compared with unexposed malnourished mice (Supplementary Fig. 8a,b). The increased weight loss in the BG-exposed malnourished mice was accompanied with increased intestinal permeability (Fig. 4b–d). When comparing jejunal gene expression of tight-junction proteins of the malnourished mice with the BG-exposed malnourished mice, we found that there is a further increase in *CLDN2* expression, both at the gene and protein level (Fig. 4b,c), and a further reduction of *CLDN4* expression in the BG-exposed malnourished mice (Fig. 4b), suggesting an exacerbation of intestinal permeability. The heightened intestinal permeability of the BG-exposed malnourished mice was functionally relevant as increased levels of FITC in the serum were measured after oral administration of FITC–dextran compared with unexposed malnourished mice and control mice (Fig. 4d). Zonulin levels in the serum have been shown previously to correlate with increased intestinal permeability in many intestinal diseases^27^, by regulating tight-junction proteins^28^, and is a biomarker for human EE^10^. We observed that BG-exposed malnourished mice have a fourfold increase of zonulin in the serum, compared with unexposed malnourished mice and control mice (Fig. 4e).

Villous blunting is a histological hallmark of human EE, and we show that BG exposure induced villous blunting, as BG-exposed malnourished mice had significantly shorter villi in the jejunum (250 μm on average) compared with the villi of unexposed malnourished mice (350 μm on average; Fig. 4f and Supplementary Fig. 9c,d). An alternative measure of villous blunting is the ratio of the villous length to crypt depth, which decreased from 4:1 in the malnourished mice to 2.5:1 in the BG-exposed malnourished mice (Fig. 4f). Given the histological differences, we assessed whether BG exposure induced small intestinal inflammation in malnourished mice. We measured *ex vivo* jejunal cytokine secretion from cultured jejunal sections and found significantly elevated interleukin (IL)-6 and monocyte chemotactic protein-1 (MCP-1) production in BG-exposed malnourished mice only (Fig. 4g). To further confirm the presence of chronic inflammation in this model, we tested the impact of BG exposure on another EE biomarker^10^,^12^, faecal calprotectin, whose levels correlate positively with intestinal inflammation^29^. The concentration of calprotectin in the faeces of BG-exposed malnourished mice was 2.5 greater than controls or unexposed mice, confirming the presence of significant intestinal inflammation (Fig. 4h). Notably, the BG exposure had no effect on TJP expression and protein levels, intestinal permeability, serum zonulin or faecal calprotectin levels in mice fed the control diet (Fig. 4b–d,g).

Given that the BG cocktail contains Gram-negative bacteria, we sought to understand the role of TLR4 signalling in the induction of villous blunting and inflammation. Five of the seven Bacteroidales and *E. coli* strains used were able to sufficiently activate TLR4 (Supplementary Fig. 9a). However, TLR4-deficient mice exposure to BG still resulted in significant blunting of the jejunal villi, along with upregulated IL-6 secretion, where MCP-1 secretion remained unchanged (Supplementary Fig. 9b–e). Furthermore, the metabolic activity of the BG bacteria are required to induce any measurable level of villous blunting or small intestinal inflammation in malnourished mice, as mice given a heat-inactivated BG did not develop these characteristic EE features (Supplementary Fig. 9c–e).

Unexpectedly, neither the administration of the Bacteroidales species alone nor the *E. coli* species alone were sufficient to trigger the growth stunting, inflammation or villous blunting characteristic of EE as seen with the combined BG (Supplementary Fig. 8a–e). This implies that the Bacteroidales species and *E. coli* isolates work synergistically to impart their effect on the small intestine. We also assessed five additional bacterial mixtures including a *Ruminococcus* mix, *Clostridium* mix, *Prevotella* mix and Peptostreptococcaceae mix, along with VSL#3, a commercial probiotic, and found that only exposure to BG was able to exacerbate the effects of malnourishment and result in the development of EE features (Supplementary Fig. 8a–d and Supplementary Table 4).

### The BG challenge alters microbial colonization patterns

**Figure 5:**
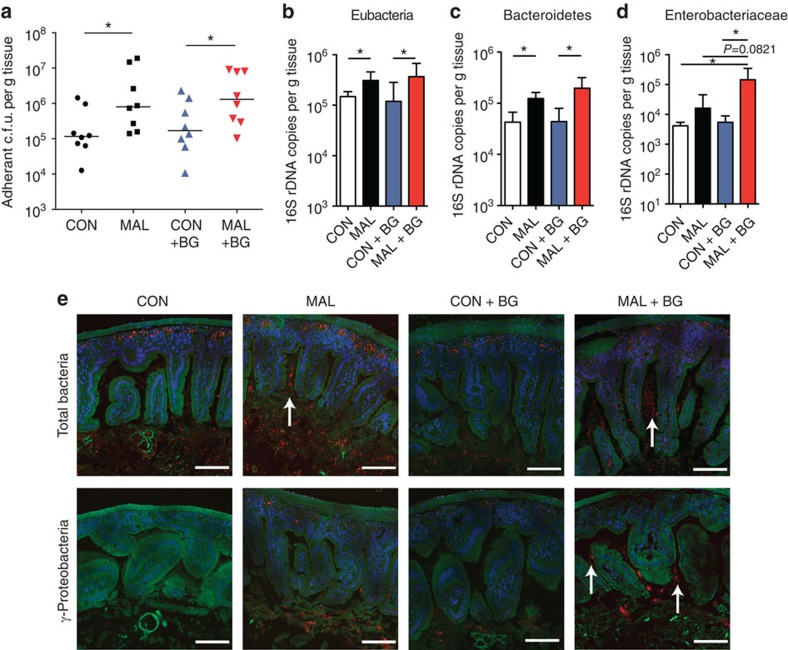
Assessing the abundance, colonization and localization of small intestinal microbiota in mice with or without Bacteroidales–*E. coli* oral exposure. (**a**) The total adherent anaerobic colony-forming units (c.f.u.'s) per gram of washed jejunal tissue in BG-exposed or unexposed malnourished and control mice. Data are pooled from two independent experiments (*n*=8), and bars indicate median values (*P<0.05, Mann–Whitney *U*-test). Real-time qPCR analysis of (**b**) the total Eubacteria 16S rDNA copies, (**c**) mouse intestinal Bacteroidetes-specific 16S rDNA and (**d**) Enterobacteriaceae-specific 16S rDNA, in each gram of jejunal tissue. Analysis was performed on DNA extracted from washed jejunum tissue of BG-exposed or unexposed malnourished and control mice. All data in **b**–**d** are pooled from two independent experiments (*n*=8), and bars indicate the mean with s.e.m. (*P<0.05, one-way analysis of variance with *post hoc* Tukey's test). (**e**) Carnoy's-fixed jejunal tissues were probed for total 16S rDNA (Eub338) and γ-Proteobacteria-specific 16S rDNA (Gam42a) abundance using FISH. Images are representative of BG-exposed or unexposed malnourished and control mice. Actin is stained in green (488-Phalloidin), cell nuclei in blue (4,6-diamidino-2-phenylindole) and bacteria in red (Eub338 and Gam42a). Scale bars, 100 μm length, and white arrows point towards the presence of tissue-associated bacteria or bacteria within the villi. CON, control; MAL, malnourished.

We next analysed the bacterial colonization levels in the small intestine of mice on each diet, with or without exposure to the BG. We found malnourished mice and BG-exposed malnourished mice had a significant increase in adherent, cultivable anaerobic bacteria in the jejunum compared with control mice (Fig. 5a). Quantification of 16S rRNA levels in jejunal tissue showed a similar trend to the culture-based analysis, as mice fed a malnourished diet had more bacteria associated with the small intestinal tissue (Fig. 5b). Surprisingly, BG-exposed malnourished mice did not have appreciably more bacteria adhering to the small intestine in comparison with the malnourished, unexposed group, despite the extensive villous blunting and increase in intestinal inflammation (Fig. 5b). Bacteroidetes 16S rRNA expression analysis showed similar levels of Bacteroidetes (MIB) in BG-exposed and unexposed malnourished mice, which was greater than the control groups (Fig. 5c). Enterobacteriaceae 16S rRNA expression analysis showed significantly more adherent Enterobacteriaceae in the small intestine of BG-exposed malnourished mice compared with unexposed malnourished mice, and importantly this was not seen in BG-exposed control mice (Fig. 5d). These data suggest that mice given a malnourished diet are more susceptible to colonization of bacteria in the small intestine, especially those from the Enterobacteriaceae family.

To distinguish the localization of bacteria in the small intestine and visually confirm the increase in tissue-associated bacteria in the small intestine, histological slices of the tissues were subjected to analysis using fluorescence *in situ* hybridization (FISH). By using a universal bacterial DNA probe, a larger number of stained bacteria could be visualized between the villi and associated with the epithelium of the jejunum of malnourished mice (Fig. 5e). Even more prominent was the increase of tissue-associated bacteria after the administration of BG to malnourished mice, where large numbers of bacteria were found to reside along the epithelium and deep within the villi (Fig. 5e). Using γ-Proteobacteria-specific probes, the increase in tissue-associated bacteria in this phylum was most striking in the BG-exposed malnourished mice (Fig. 5e). Complementing the pyrosequencing and qPCR data, FISH analysis also revealed an expansion of Bacteroidetes in the jejunal samples, a decrease in the amount of Firmicutes and more adherent bacteria along the epithelium in all groups (Supplementary Figs 10 and 11). Overall, of the groups analysed by FISH, γ-Proteobacteria penetrated deep into the crypts more than species from the Firmicutes or Bacteroidetes phyla (Fig. 5e and Supplementary Figs 10 and 11). Of note, this increase in adherent bacteria coincides with an increased expression of antimicrobial peptides or defence molecules by the host epithelium in BG-exposed malnourished mice, as matrix metalloproteinase 7 is significantly elevated, along with resistin-like molecule-β, and trends to higher expression of REG3-γ and cryptdin (Supplementary Fig. 12). These data indicate that the observed increased bacterial load adhering to the intestinal epithelium is not because of decreased antimicrobial peptide production in the jejunum.

### Influx of IELs contribute to inflammation after BG exposure

**Figure 6:**
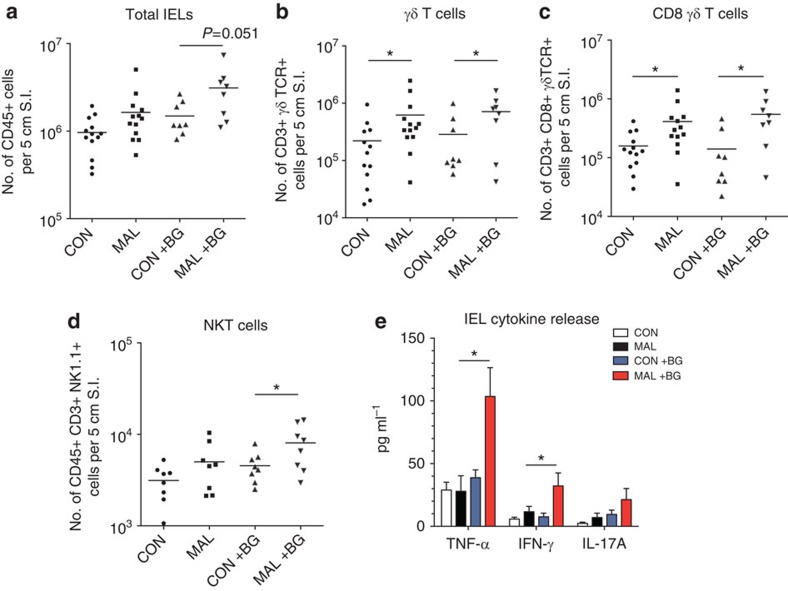
Flow cytometry and cytokine secretion analysis of small intestinal IELs. The total number of live (**a**) CD45+ cells, (**b**) CD45+CD3+γδTCR+ cells, (**c**) CD45+CD3+CD8+γδTCR+ cells and (**d**) CD45+CD3+NK1.1+ cells isolated from the upper 5 cm of the small intestine (duodenum) in BG-exposed and unexposed mice on each diet. Data from the unexposed malnourished and control mice are representative of three independent experiments pooled (*n*=13). Data from the BG-exposed malnourished and control mice are representative of two independent experiments (*n*=8). Bars indicate the mean values (*P<0.05, one-way analysis of variance (ANOVA) with *post hoc* Tukey's test). (**e**) Concentrations of TNF-α, IFN-γ and IL-17A after stimulation of cultured IELs for 48 h. Data are pooled from two independent experiments (*n*=8 per group). Bars indicate the mean values (*P<0.05, one-way ANOVA with *post hoc* Tukey's test). CON, control; MAL, malnourished.

Owing to the presence of intestinal inflammation and villous blunting observed after BG exposure, we investigated the impact of diet and BG on the intestinal immune cell populations. We hypothesized that shifts in lymphocytes in the small intestine could be driving inflammation, and, consequently, the barrier defect observed in the malnourished mice. We first isolated lymphocytes from the small intestine and discovered that the malnourished diet led to increase in numbers of intraepithelial lymphocytes (IELs) in the duodenum compared with control mice, and that this difference was greater in BG-exposed malnourished mice, although not statistically significant (one-way analysis of variance, *P*=0.051; Fig. 6a). Within the duodenum IEL compartment, there was a fivefold increase in the numbers of γδ T cells in the malnourished mice and a similar fivefold increase in BG-exposed malnourished mice, compared with controls (Fig. 6b). The majority of the influx of γδ T cells consisted of CD8+γδ T cells in both BG-exposed and unexposed malnourished mice (Fig. 6c and Supplementary Fig. 8a). CD4+ and CD8+ αβ T-cell numbers in the IEL compartment were not significantly different between groups; however, abundance of double-positive CD4+CD8+ γδ T cells, counter-intuitively, decreased in abundance after BG was administered (Supplementary Fig. 13b–d). Much of this influx of IELs in the duodenum was driven by the malnourished diet alone; however, NKT-cells were significantly more abundant in BG-exposed malnourished mice compared with malnourished mice (Fig. 6d). Cultured IELs from the duodenum of BG-exposed malnourished mice secreted significantly greater quantities of tumour necrosis factor alpha (TNF-α) and interferon gamma (IFN-γ), as well as a trend for elevated IL-17A compared with unexposed malnourished and control mice, after T-cell specific stimulation (Fig. 6e). These findings suggest that the malnourished diet is sufficient to induce the IEL influx; however, heightened cytokine production by these cells is only triggered with exposure to the BG, and this may contribute to the inflammation and villous blunting observed specifically in the BG-exposed malnourished mice.

### Impact of malnutrition and BG on enteric infection

**Figure 7:**
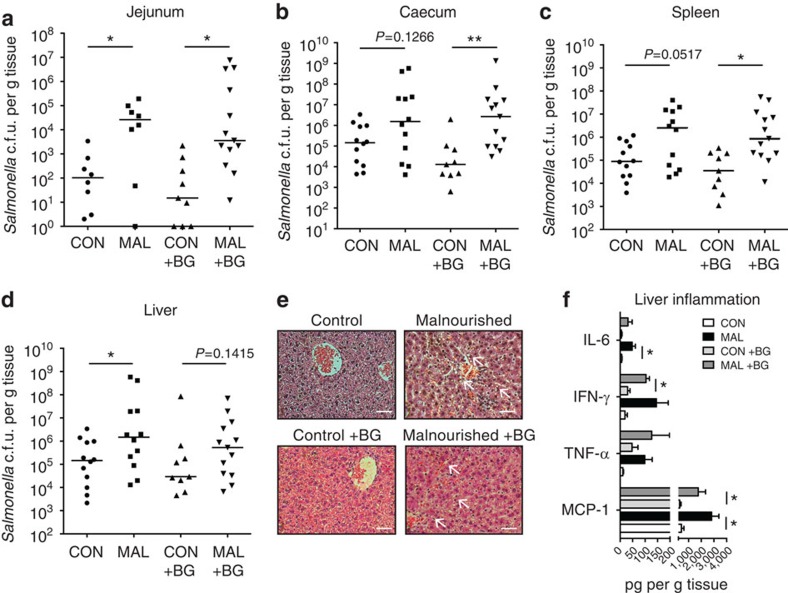
Systemic colonization and tissue burden of *S.*Typhimurium in malnourished and control mice. Total c.f.u. of *Salmonella* per gram of tissue was assessed 3 days post infection in the (**a**) jejunum, (**b**) caecum, (**c**) spleen and (**d**) liver in BG-exposed and unexposed malnourished and control mice fed the diet for 3 weeks. Data are pooled from two independent experiments (*n*=8–13 per group), and bars indicate median values (*P<0.05, Mann–Whitney *U*-test). (**e**) H&E-stained slices of the livers of BG-exposed and unexposed malnourished and control mice 3 days post *Salmonella* infection. Scale bars, 100 μm in length, and white arrows point towards pathological features. (**f**) Concentrations of IL-6, MCP-1, TNF-α and IFN-γ per gram of liver tissue 3 days post *Salmonella* infection as measured by cytokine bead array. Data are pooled from two independent experiments (*n*=8–13 per group). Bars indicate the mean with s.e.m. (*P<0.05, Student's *t*-test). CON, control; MAL, malnourished.

To understand the impact of malnutrition and BG exposure on enteric infection, we utilized the well-studied enteric pathogen *Salmonella enterica subsp. typhimurium* (*S.* Typhimurium) as its colonization dynamics in the small intestine are well known^30^. Furthermore, its greatest burden is in regions of the world where children are also affected by EE^31^. In mice fed a standard diet and orally infected with *S.* Typhimurium, very few *S.* Typhimurium colony-forming units (c.f.u.'s) were detected in the small intestine (Fig. 7a). In contrast, mice infected orally after being fed a malnourished diet for 3 weeks post weaning had a 3 log fold increase in the burden of *S.* Typhimurium adherent to the jejunum 3 days post infection in BG-exposed and unexposed mice (Fig. 7a). There was a trend towards increased *S.* Typhimurium colonization in the caecum of malnourished mice, but *S.* Typhimurium colonization significantly increased in the caecum of BG-exposed malnourished mice compared with controls (Fig. 7b). Malnourishment led to increased systemic burdens of *S.* Typhimurium with significantly increased colonization in the liver and spleen (Fig. 7c,d). The liver of the infected malnourished mice exhibited striking gross and histological changes. Histological examination showed greater levels of cell necrosis and the presence of fatty lipid droplets (Fig. 7e). Higher levels of inflammatory cytokines, including IL-6, IFN-γ, TNF-α and MCP-1, were released in the livers of BG-exposed and unexposed malnourished mice 3 days post-infection compared with control, infected mice (Fig. 7f). Overall, malnourished mice were more susceptible to *S*. Typhimurium infection, and this increased susceptibility was maintained on exposure to BG, exhibiting higher bacterial burdens in the intestine, while also invading systemically to greater levels, and inducing a prominent pathological change in the liver.

## Discussion

These results indicate that a malnourished diet profoundly alters the small intestinal ecosystem, permitting a greater probability of colonization from environmentally acquired microbes. We have identified a set of commensal Bacteroidales and *E. coli* strains that, only in combination with a malnourished diet, are able to reproduce the features of human EE in young mice. To date, it has been unclear which species of bacteria, if any, contribute to EE phenotypes^32^. This observed impact of the Bacteroidales–*E. coli* challenge (BG) is consistent with our hypothesis and previous literature suggesting a microbial aetiology for EE^15^, where environmental ingestion of particular microbes can trigger EE and worsen the impact of childhood malnutrition.

A number of epidemiological and microbiological studies indicate that increased oral bacterial exposure early in life (due to poor environmental sanitation) is behind the aetiology of EE^17^,^33^,^34^, and in a number of these cases, children exhibit small intestinal bacterial overgrowth^35^,^36^. Notably, contamination of the household environment with *E. coli* and Bacteroidales species was common and correlated with increased intestinal permeability, as well as growth rate deficiencies in rural areas of Zimbabwe and Bangladesh^17^,^33^. However, this observation remains a correlative one and other microbes could be involved. In the limited studies assessing the small intestine of malnourished children with EE features, breath test and culture-based analysis indicated the presence of small intestinal bacterial overgrowth^37^ and Gram-negative bacteria colonizing in the duodenum^38^, consistent with our observations in malnourished mice. To date, no studies have performed high-throughput sequencing analysis on the small intestine of children with EE, thus we do not have information on the prevalence of Bacteroidales and *E. coli* in these patients, or whether other Gram-negative bacteria may be involved.

By utilizing FISH, we were able to visualize an increased abundance of tissue-adherent bacteria triggered by the diet and exacerbated with exposure to Bacteroidales*–E. coli* mixture, this observation is also in agreement with other forms of chronic intestinal inflammation^39^, suggesting that the microbiota drive the inflammation. More specifically, the qPCR and FISH data indicate that the Enterobacteriaceae abundance was substantially higher in the Bacteroidales*–E. coli*-exposed malnourished mice. These data indicate that the BG exposure combined with a malnourished diet aids in the ability of Enterobacteriaceae species to associate or adhere to the mucosa. Also, the increased abundance of Bacteroidales and Enterobacteriaceae after BG exposure suggests that the malnourished diet promotes the growth potential of these microbes in the small intestine. The inflammatory-driving properties of Enterobacteriaceae species have been well documented, and human commensal *E. coli* species are linked to the development of intestinal inflammation in susceptible hosts^40^,^41^. Unexpectedly, when administered separately, neither Bacteroidales species nor *E. coli* were sufficient to induce EE features, indicating that these species could work in synergy to alter the small intestinal ecosystem enough to promote the dysbiotic changes in function observed. This result was supported by recent data that demonstrated that IgA-linked microbiota from severely malnourished children were sufficient to transfer the Kwashiokor phenotype to germ-free mice (severe inflammation and villous blunting), and Enterobacteriaceae that were essential, however, were not able to induce villous blunting alone without the other bacteria in the mixture^42^. Interestingly, this mixture of bacteria included two of the same species of Bacteroidales strains to induce EE features in the BG mix, and also an *E. coli* strain. Despite their symbiotic capabilities^43^, Bacteroidales species also possess the ability to be pathobionts and drive inflammation in the intestine of susceptible hosts^44^,^45^,^46^; however, in our model, they are not pathogenic unless combined with commensal *E. coli.*

The metabolomic analysis of the small intestine revealed for the first time that malnutrition leads to drastic shifts in small intestinal bile acid pool. Specifically, a reduction in tauro-conjugated bile acids was observed in malnourished mice, which are important for fat and nutrient uptake in the small intestine^47^, and based on previous reports in patients with tauro-conjugated bile acid deficiency, also could be linked to the observed alterations the luminal bioavailability of the fat-soluble vitamins A, D and E^48^. Bile acid changes could also be a factor into the observed resemblance of the microbiota in the malnourished duodenum to that of the ileum. Many studies report that bile acids are one of the major mechanisms to restrict bacteria proliferation in the upper small intestine^49^, and can impact host–microbial interactions during intestinal inflammation^50^. Thus, the shift in bile acids could be the mechanism that permits for the increased colonization of commensal Bacteroidales and *E. coli* strains in the small intestine, and may also be behind the ability of *S.* Typhimurium to proliferate in the malnourished small intestine to higher abundance. The infection with *S.* Typhimurium highlighted the observations from the BG exposure that malnutrition induces an increased proliferative capacity and colonization potential of Gram-negative bacteria in the small intestine. The increased ability for *S.* Typhimurium to invade systemically also gives biological relevance to the increase in intestinal permeability observed pre-infection in malnourished mice^51^.

The expansion of duodenal IELs observed in malnourished mice seems contradictory to the literature, since protein malnutrition is known to lead to depressed immune function^52^. However, in the limited studies where biopsies of the small intestine were taken from children with EE features, there is evidence of a marked infiltrate of lymphocytes to the epithelium, notably γδ T cells and activated CD8+ T cells^53^, an observation consistent with our results in this model. While BG exposure did not alter total numbers of CD8+γδ T cells in malnourished mice, IELs isolated from BG-exposed malnourished mice secreted more inflammatory cytokines after stimulation, and this increase in inflammatory potential after stimulation may be driving the histopathology observed after BG exposure. γδ T cells also proliferate to high numbers in coeliac disease, which shares similar histopathological characteristics in the small intestine as EE^54^, as well as small intestinal microbiota dysbiosis^55^.

The malnourished diet we utilized was similar to the regional basic diet used to induce moderate protein energy malnutrition in mice in previous studies^56^,^57^,^58^. However, these studies did not characterize the impact of the diet on the microbiota, metabolism and intestinal immune cells, or assess the impact of oral challenge of defined set of bacteria on EE features. In doing these analyses, we utilized a holistic approach to demonstrate novel observations in how malnutrition in mice alters the small intestinal microenvironment and utilized these data to develop this model. Furthermore, this mouse model showed increased concentrations of zonulin in serum and in faecal calprotectin, both proposed biomarkers of EE in humans^10^,^12^, showcasing the relevance of this model to human disease.

In summary, we show that an orally administered cocktail of specific bacteria are able to reproducibly replicate features of EE only in mice fed a malnourished diet. Utilizing this model, we were able to achieve for the first time an in-depth characterization of the impact of malnutrition on the microbiota, metabolism and immune system in the mammalian small intestine. Future studies will utilize this murine model to gain a deeper understanding of the pathophysiological nature of EE. Furthermore, this model will allow further studies testing the impact of early-life interventions to reverse EE induction.

## Methods

### Animal studies

All animal work was done according to the Canadian Council on Animal Care guidelines by utilizing protocols that were approved for use by the Animal Care Committee at the University of British Columbia. Three-week-old female C57BL/6 mice or TLR4−/− mice were ordered for each experiment (Jackson Laboratory, Bar Harbor, ME) and housed in a barrier animal facility at the University of British Columbia with a 12-h light–dark cycle. On arrival, mice were randomized and housed into separate groups (4–5 per cage), which were either fed a malnourished diet moderately low in protein (7%) and fat (5%) or an isocaloric control diet with 20% protein and 15% fat, similar to one used in previous studies to induce protein malnutrition^56^ (Research Diets, New Brunswick, NJ; Supplementary Table 1). The chow was irradiated before use and mice were given the diet *ad libitum* throughout experiments.

### RNA isolation and cDNA synthesis

Approximately 1 cm of the jejunum was excised and immediately submerged in RNA*later* (Qiagen, Valencia, CA) and stored at 4 °C overnight and then at −80 °C for subsequent RNA extraction. The RNA of the tissue was extracted using RNeasy Mini kit (Qiagen) according to the manufacturer's instructions. RNA concentration and purity were determined using a NanoDrop ND-1000 (NanoDrop Technologies, Wilmington, DE, USA), and reverse transcription was completed with the Quantitect reverse transcription (RT) kit (Qiagen) utilizing a total of 1 μg RNA as template for the reaction.

### Real-time qPCR for host gene expression

Real-time qPCR analysis for host gene expression was performed utilizing Quantitect SYBR-Green Mastermix (Qiagen) by using primers listed in Supplementary Table 4. PCR was performed in 10 μl reaction volumes on an Applied Biosystems 7500 machine and cycles consisted of 95 °C for 15 min and 40 cycles of 95 °C for 15 s, 60 °C for 30 s and 72 °C for 30 s. Glyceraldehyde phosphate dehydrogenase was found to be an appropriate endogenous control and was used for normalization. Relative expression was calculated using the ΔΔ*C*(*t*) method relative to the control mice.

### Real-time qPCR analysis for bacterial abundance

The assessment of bacterial abundance was performed using Quantitect SYBR-Green Mastermix (Qiagen) and group-specific primers for 16S rRNA (Supplementary Table 5). PCR was performed on an Applied Biosystems 7500 machine. The abundance of 16S rRNA in the small intestinal sample was determined by comparing the *C*_T_ values to the values generated by standard curves. The standard curves were developed from applying the group-specific primers to DNA purified from cultured American Type Culture Collection (ATCC) (Manassas, VA, USA) strains of bacteria with a known value of 16S copies per nanogram of DNA. Results were expressed in total 16S copies per gram of tissue extracted.

### FITC–dextran uptake assay

To directly assess intestinal permeability *in vivo*, mice were gavaged with 80 mg ml^−1^ of 4 kDa FITC–dextran (Sigma-Aldrich) at a volume of 150 μl after food deprivation for 4 h. Four hours post inoculation, serum was collected from mice postmortem and measured for FITC concentration using a plate reader (Tecan, Maennedorf, Switzerland). FITC was measured against a standard curve of serially diluted FITC–dextran, and the plate was read with the excitation of 485 nm and emission of 530 nm.

### Histology

Intestinal sections 2 cm in length from the duodenum, jejunum and ileum of mice were collected and immediately placed in 10% buffered formalin overnight at room temperature. Paraffin-embedded tissues were cut into 5 μm slices and stained with haematoxylin and eosin (H&E) using standard techniques. H&E-stained tissues were visualized under a light microscope and villous length, and crypt depth of each crypt and villous of the tissue were enumerated using Axiovision version 4.6 software. Livers were excised, cut and stained with H&E in a similar manner as stated above and visualized under a light microscope for signs of pathology. For visualizing the mucus layer, 1 cm sections of the jejunum were excised from mice, immediately submerged into methanol–Carnoy's fixative for 2 h at 4 °C and then transferred to 100% ethanol. Paraffin-embedded tissues were cut into 5 μm slices and stained with Alcian Blue-periodic acid Schiff using standard techniques.

### DNA extraction and microbiota analysis

To assess the composition of the microbiota, sections from the small intestine of malnourished or control-fed mice were homogenized using a bead-beating method (FastPrep instrument, MP Biomedicals, Solon, OH), and total DNA was extracted using a Stool DNA Extraction Kit (Qiagen). 16S rRNA gene fragments were PCR amplified with nucleotide bar-coded primer pairs 27F: 5′-AGAGTTTGATCMTGGCTCAG-3′and 510R: 5′-GWATTACCGCGGCKGCTG-3′. PCR products were gel-purified (QIAquick gel extraction kit, Qiagen). Each amplicon (100 ng) was pooled and sequenced using a 454 Titanium platform (Roche, Branford, CT).

### Bioinformatics

All sequences were processed using MOTHUR according to the standard operating as previously described^59^, accessed on 10 July 2013. Quality sequences were obtained by removing sequences with ambiguous bases, a quality read length <200 bases and/or chimeras identified using chimera.uchime. Quality sequences were aligned to the silva bacterial reference alignment and OTUs were generated using a dissimilarity cutoff of 0.03. Sequences were classified using the classify.seqs command with ribosomal database project (RDP) as reference. Inverse Simpson's diversity index was used to calculate diversity. Differences in microbial communities between groups and intestinal sites were investigated using the phylogeny-based weighted UniFrac distance metric. The Bray Curtis index was used as a measure of similarity in microbial composition. Diversity, similarity and abundance of bacterial OTUs and families were compared using the Mann–Whitney *U*-test or Student's *t*-test, and the Bonferroni correction was applied in cases of multiple comparisons.

### Metabolite extraction

Mouse small intestine digesta samples (luminal contents) were collected, weighed and homogenized in water followed by addition of acetonitrile for metabolite extraction. After vortex mixing, 30 s sonication and centrifugation, the clear supernatants were collected and dried under a gentle nitrogen gas flow. The dried metabolite residues were dissolved in 40% acetonitrile containing 0.01% formic acid, 4 μl mg^−1^ of the raw material. After 15 s vortex and 30 s sonication, each sample was further diluted 1:2 with water. Twelve-microlitre aliquots were injected for untargeted metabolic fingerprinting by UPLC–FTMS.

### UPLC–FTMS

A Waters Acquity UPLC system coupled to a Thermo LTQ-Orbitrap Velos Pro mass spectrometer was used for metabolomic analysis. The mass spectrometer was equipped with a heated electrospray ionization (ESI) source and was operated in the FT mass spectrometry (MS) scan mode with mass resolution of 60,000 full-width at half-maximum at *m/z* 400. The *m/z* detection range was 80–1,200. A Waters BEH C_18_ UPLC column (2.1 mm × 50 mm, 1.7 μm) was used for chromatographic separation with the mobile phases being water–formic acid (0.01%; solvent A) and acetonitrile–formic acid (0.01%; solvent B). The binary solvent elution gradient was 5–40% B in 6 min and 40–100% B in 15 min. The mobile phase was kept at 100% B for 2 min before column equilibration with 5% B for 4 min between injections. The column temperature was 50 °C and the flow rate was 0.3 ml min^−1^. Two liquid chromatography (LC)–MS runs per sample were performed in the (+) and (−) ion detection modes, respectively. Lock mass calibration was applied to ensure mass accuracy throughout LC–MS runs.

### Data processing

The positive and negative ion UPLC–FTMS data sets were converted to the mzXML files using the MSCovert tool (ProteoWizard) and processed using the XCMS package (http://metlin.scripps.edu/xcms/) in the R platform (http://www.r-project.org/). For each data set, peak detection and integration were performed using the centWave algorithm^60^. Retention time shift correction was achieved considering at least 200 peak groups. After two iterations of peak grouping, imputation of missing data was performed by returning to the raw spectral data and integrating the areas of the missing peaks using the ‘fillPeaks' algorithm. Finally, a data matrix was generated from each UPLC–MS data set and imported into Microsoft Excel. After peak de-isotoping and removal of the significant background noise signals observed in each UPLC–MS blank run, the data were saved as two-dimensional (*m/z* retention time versus peak area) matrices amenable to subsequent statistics and further data analysis.

### Metabolic pathway analysis

The resulting *m/z* values after data processing were searched against the METLIN database (http://metlin.scripps.edu/metabo_batch.php?&return=yes) for metabolite identification. The metabolic pathways that the identified metabolites were involved in were matched using the Kyoto Encyclopedia of Genes and Genomes pathway tool, permitting the over-representation analysis to determine over-represented pathways in each treatment group. Multivariate statistical analysis (random forest, PCA) and over-representation analysis were carried out using the Metaboanalyst version 2.5 software (http://www.metaboanalyst.ca/MetaboAnalyst/) based on recommendations from previous published protocols^61^. The data from the positive and negative ion mode detection were treated separately in the statistical analysis. In the analysis, missing values were assumed to have been below the level of detection in the parameters. Each data set was normalized to the median of each of the observed peaks, following a log transformation of the data. The Welch's two-sample *t*-test was used to determine significant changes between groups (*P*<0.05; fold change >2).

### Vitamin-targeted metabolomics

The samples were analysed by UPLC–MRM (multiple-reaction monitoring)/MS on a Dionex UltiMate 3400 RSLC system coupled to an AB Sciex 4000 QTRAP triple-quadrupole mass spectrometer equipped with an electrospray ionization source. The standard substances of vitamin A (retinal, retinol and retinoic acid), B1 (thiamine), B2 (riboflavin), B3 (niacinamide), B6 (pydidoximine, pyridoxine, pyridoxal and pyridoxal-mono-phosphate), B7 (biotin), B9 (folic acid), D2, D3, E (α-tocopherol, δ-tocopherol and δ-tocotrienol), K1 and K2 were purchased either from Sigma-Aldrich or from Cayman Chemicals Inc. The MRM transitions of individual analytes were optimized by direct infusion of a standard solution of each compound into the MS instrument. Each sample was added with a methanolic butylated hydroxytolueune (BHT) (2 mg ml^−1^) solution at a ratio of 15 μl mg^−1^ of the small intestine digestate. Vitamins were extracted by homogenizing the samples at a shaking frequency of 30 Hz for 1 min twice using a Retsch MM400 mixer mill and with the aid of two 3-mm stainless steel metal balls, followed by 5-min sonication in an icy water bath. The samples were then centrifuged in a microcentrifuge at 12,500 r.p.m. and 4 °C for 10 min. A 300-μl aliquot of the supernatant was transferred into a 3-ml borosilicate glass test tube and mixed with 300 μl of water and 900 μl of hexane. After 1 min vortex mixing, the tubes were centrifuged at 4,000 r.p.m. and 10 °C in a Beckman R22 centrifuge to separate the supernatant organic phase from the lower aqueous phase. The supernatants were carefully pipetted out to another sets of 3-ml test tubes. The fat-soluble vitamins were further extracted from the aqueous phase with 900 μl of hexane two more times. After liquid–liquid extraction, the pooled organic phase for each sample was dried down in a speed-vacuum concentrator at room temperature. The dried residue was reconstituted in 100 μl of ethanol. A 20-μl aliquot was injected for quantification of the fat-soluble vitamins by LC–(+)ESI–MRM/MS on Waters BEH C18 (2.1 × 50 mm, 1.7 μm) UPLC column and with 0.1% formic acid in water and acetonitrile as the mobile phase for binary solvent gradient elution. An efficient elution gradient was 50–100% B in 10 min. The column temperature was 50 °C and the flow rate was 300 μl min^−1^. The aqueous phases were loaded onto reversed-phase polymeric HLB cartridges (60 mg per 1 ml, Waters Inc.), which have been activated with 1 ml of methanol and equilibrated with 1 ml of 50% methanol before use. Under a 5-inch Hg vaccum, the flow-through fractions were collected, and the resins were washed with 1 ml of 50% methanol with the flow-through fractions collected. The pooled flow-through fractions were dried in a nitrogen evaporator at 30 °C. The residue from each sample was reconstituted in 100 μl of 2% methanol. A 20-μl aliquot was injected for quantification of the water-soluble vitamins by UPLC–MRM/MS with (+) or (−) ESI and on a Waters BEH C18 (2.1 × 150 mm, 1.7 μm) UPLC column and using 0.01% formic acid in water and methanol as the mobile phase for binary solvent gradient elution. The efficient elution gradient was 2% B for 0.5 min and 2–50% B in 8 min. The column temperature was 30 °C and the flow rate was 250 μl min^−1^. The concentrations of all the detected vitamins were calculated from the standard calibration curves of individual vitamins, which were prepared with the use of their authentic compounds.

### Bile acid-targeted metabolomics

Each sample was homogenized in LC–MS grade water at a ratio of 150 μl per 10 mg raw material and with the aid of 5-mm stainless steel metal balls. Bile acids were extracted by addition of acetonitrile at a ratio of 350 μl per 10 mg raw material followed by vortexing and sonication (1 min) in an ice–water ultrasonic bath. The samples were centrifuged. Twenty microlitres of the supernatants were precisely taken out and mixed with a predefined mix of 14 deuterium-labelled bile acids as the internal standards. The mixtures were subjected to phospholipid-depletion solid-phase extraction according to a validated protocol for sample cleanup and bile acid enrichment^62^. The flow-through fractions were collected and then dried under a gentle nitrogen flow. The dried residues were dissolved in 200 μl of 50% methonal. Ten microlitres of residues were injected for quantification by UPLC–MRM/MS. A Dionex UPLC system was connected to an AB Sciex 4000 QTRAP mass spectrometer that was operated in the negative ion MRM mode and with electrospray ionization. UPLC separation was carried out on a 15-cm-long C-18 UPLC column with water–acetonitrile–formic acid as the mobile phase for binary gradient elution using a developed and validated protocol for comprehensive analysis of bile acids in biological samples (Han *et al*., manuscript submitted to Analytical Chemistry). The column temperature was 45 °C and the flow rate was 0.35 ml min^−1^. Forty-five bile acids (including the 19 targeted bile acids) were involved in the quantification by UPLC/scheduled MRM/MS. Concentrations of the detected bile acids were calculated with internal standard calibration from the linearly regressed standard calibration curves of individual bile acids. The lower limits of quantification were 0.08 nmoles per mg for all the bile acids.

### SCFA analysis

Small intestinal samples were weighed and combined with 25% phosphoric acid, vortexed and centrifuged until a clear supernatant was obtained. Supernatants were submitted for gas chromatography (GC) analysis to the Department of Agricultural, Food and Nutritional Science of the University of Alberta. Samples were analysed as previously described^63^ with modifications. Briefly, samples were combined with 4-methyl-valeric acid as an internal standard and 0.2 ml was injected into the Bruker Scion 456 gas chromatograph, using a Stabilwax-DA 30 m × 0.53 mm × 0.5 μm column (Restek, Bellefonte, PA). A standard solution containing acetic acid, propionic acid, isobutyric acid, butyric acid, isovaleric acid, valeric acid and caproic acid, combined with internal standard, was injected in every run. The programmed temperature vaporizing (PTV) injector and flame ionization detector (FID) detector temperatures were held at 250 °C for the entire run. The oven was started at 80 °C and immediately ramped to 210 °C at 45 °C per min, where it was held for 5.11 min. Total run time was 8.00 min. Helium was used at a constant flow of 20.00 ml min^−1^.

### Bacterial strains

The *S.* Typhimurium strain SL1344 was used, as this strain is virulent to mice and resistant to streptomycin^64^. Colonies of SL1344 were grown on Luria-Bertani (LB) agar with 100 μM streptomycin. Colonies were selected and inoculated for growth in LB broth at 37 °C with shaking. All commensal bacterial strains that are used in our study (Supplementary Table 4) were of human origin, and were provided to us by Emma Allen-Vercoe (University of Guelph) or ordered from the ATCC and DSMZ culture collections (Leibniz Institute, Germany). Commensal bacteria strains were all grown on fastidious anaerobe agar (FAA; LabM) in anaerobic conditions.

### Bacterial cocktail preparation and inoculation

In an anaerobic chamber, bacterial cultures from frozen stock were first plated on FAA, and subsequently pure cultures were selected and mixed together at a 1:1 ratio in sterile, reduced PBS. Bacterial mixtures in PBS were removed from the anaerobic chamber and were immediately transported to the animal facility to carry out gavage experiments. The volume of the mixture received per mouse was 100 μl at a concentration of 10^9^ cells per ml. The concentration of the mixture in cell per ml was carried out using a ultraviolet spectrometer, and gavage doses were confirmed after by back-titering the inocula on FAA. In experiments using heat-inactivated bacteria, the bacterial mixture was incubated for 2 h at 80 °C and plated on FAA to confirm the absence of growth.

### Intestinal epithelial cell (IEC) and IEL isolation

The upper 5 cm of the small intestine of mice was excised, attached fat and Peyer's patches were removed, and tissues were cut longitudinally to further remove luminal contents by washing with ice-cold PBS. Epithelial cells were isolated using a PBS buffer containing 1 mM EDTA, 1 mM dithiothreitol and 5% foetal bovine serum (FBS), shaking at 37 °C for 10 min. This supernatant was filtered and combined with RPMI 1640 and centrifuged at 1,500 r.p.m. to isolate the IECs. The intact intestinal tissue was resuspended in additional PBS buffer containing 1 mM EDTA, 1 mM dithiothreitol and 5% FBS, shaking at 37 °C for 20 min to isolate IELs. Lymphocytes were further purified using a 40% Percoll gradient and were resuspended in RPMI 1640 with 5% FBS.

### Flow cytometry

Purified lymphocytes were counted using an automatic cell counter (Countess, BD Biosciences). Cells were stained with a 1/200 dilution of fluorochrome-conjugated antibodies against CD45 (clone 30F-11), CD3ɛ (eBio500A2), CD4 (RM4–5), CD8 (53-6.7), NK1.1 (PK136) and γδ TCR (eBioGL3) (eBioscience), and their populations were analysed by an LSR II flow cytometer (BD Biosciences) using software packages from CellQuest and FlowJo version 8.7.

### Western blotting

Total IECs were lysed with MP-40 and protease inhibitor cocktail (Roche Diagnostics, Basel, Switzerland) for 10 min on ice. The resulting lysis solution was centrifuged at 13,000 r.p.m. (16,360*g*) for 10 min, and the supernatant was collected. The protein concentration of each sample supernatant was quantified using a bicinchoninic acid assay (Sigma-Aldrich). Equal concentrations of the samples were electrophoresed through a 12% SDS–polyacrylamide gel and transferred onto nitrocellulose membrane (Millipore, Darmstadt, Germany). Membranes were probed with 1/500 dilutions of anti-*CLDN2* and anti-actin primary antibodies (Invitrogen, Carlsbad, CA) and then a 1/1,000 dilution of a secondary anti-rabbit/mouse-horseradish peroxidase antibody.

### Faecal calprotectin determination

Faecal content of mice was collected, weighed, homogenized in PBS, centrifuged at 13,000 r.p.m. (16,360*g*) and the resulting supernatant was stored at −80 °C. The concentration of calprotectin in faeces was performed on the faecal supernatants using a S100A8/S100A calprotectin enzyme-linked immunosorbent assay (ELISA) kit as per manufacturer's instructions (Hycult Biotechnology). The resulting concentration of calprotectin was determined by assessing the OD 450 nm using a plate reader (Tecan), comparing with a standard curve of known concentrations of calprotectin.

### Total IgA determination

Total IgA concentrations were measured in the faeces and small intestinal luminal content using a commercially available ELISA kit (eBioscience, cat. no. 88-50450-22). Faecal and small intestinal homogenates were weighed and diluted to 1:1,000 for the assay. Sample concentrations were determined by assessing the OD 450 nm using a plate reader (Tecan), compared with a standard curve, and normalized to weight of the faecal content.

### Serum zonulin determination

Serum zonulin concentrations were measured using a zonulin ELISA kit (Abcam, Cambridge, UK) as per the manufacturer's instructions. Sample concentrations were determined by assessing the OD 450 nm using a plate reader (Tecan), compared with a standard curve. This assay only identifies the active, uncleaved form of zonulin in the sera of mice.

### Infection models of mice

A frozen stock of *S.* Typhimurium (SL1344) was first plated on LB agar with 100 μM streptomycin and a single colony was subsequently inoculated into LB broth and grown, with shaking, for 18 h at 37 °C. Using this culture, mice were infected by oral gavage with 100 μl of 5 × 10^6^ cells per ml. Mice were monitored daily throughout infection as per the animal care protocol and euthanized 3 days post infection.

### *S.* Typhimurium c.f.u. and cytokine determination

At 3 days post infection, tissues were collected from infected mice and placed into 1 ml of sterile PBS with complete EDTA-free protease inhibitor cocktail (Roche Diagnostics) at a final concentration as recommended by the manufacturer. All tissues were weighed, then homogenized with a MixerMill 301 bead miller (Reutsch) for 2 min at room temperature. Tissue homogenates were serially diluted in PBS, plated on LB agar with 100 μM streptomycin and incubated overnight at 37 °C. Bacterial colonies were enumerated the following day to determine the c.f.u. per gram of tissue. To determine the cytokine concentrations, tissue homogenates were centrifuged twice at 15,000 r.p.m. (21,660*g*) for 20 min at 4 °C to remove cell debris and the resulting supernatants were aliquoted and stored at −80 °C. Cytokine levels in the homogenates were measured using a Cytometric Bead Array Mouse Inflammation Kit (BD Biosciences), according the manufacturer's recommendations.

### IEC and IEL cytokine quantification analysis

For *ex vivo* quantification of cytokine secretion in the small intestinal tissues, jejunal slices of tissue were washed with complete tissue culture media (RPMI, 10% FBS, 1% glutamine and 1% of 1:1 penicillin:streptomycin) and cultured in 1 ml of the same media in 24-well plates for 24 h at 37 °C, 5% carbon dioxide. The supernatants were screened for the production of inflammatory cytokines using a CBA flex set (BD Biosciences). For analysis of IEL-secreted cytokines, isolated lymphocytes were cultured in complete tissue culture media 24-well plates at a density of 1.0 × 10^6^ cells per ml. Cells were stimulated with 4 ng ml^−1^ of rIL-2 and 1 μ ml^−1^ of anti-CD3/CD28, and were incubated for 48 h at 37 °C, 5% carbon dioxide. The resulting supernatants were used to determine the amount of TNF-α, IFN-γ (BD Biosciences, cat. nos. 560478 and 558258) and IL-17A (eBioscience, cat. no. 88-7371-88) using murine-specific ELISA kits relative to a standard curve and according to the manufacturer's recommendations.

### Fluorescence *in situ* hybridization

A 1-cm section of the jejunum was excised from mice, immediately submerged into methanol–Carnoy's fixative for 2 h at 4 °C, and then transferred to 100% ethanol. Fixed tissues were paraffin embedded, cut with a microtome to a thickness of 6–10 μm and transferred to glass slides. The slides were let dry overnight and the tissues were then fixed with 4% paraformaldehyde for 10 min at room temperature before permeabilization with a solution of 1 mg ml^−1^ lysozyme from Eggwhite at 37 °C for 1 h. The tissues where then pre-incubated for 1 h at 42 °C in hybridization buffer (20 mM Tris-HCl, pH 8.0, 0.9 M NaCl, 0.01% SDS and formamide, depending on the probe; Sigma-Aldrich), and then the slides were let dry at 37 °C and incubated overnight at 42 °C with the respective probe linked to Alexa 555 (Invitrogen) in the corresponding hybridization buffer at a probe concentration of 50 nM. The following probes were used: all-Bacterial probe (Eub338), Firmicutes (equimolar mixture of LGC354A, B and C), Bacteroidetes (Bac303) and Gamma-Proteobacteria (Gam42a). Details on the probes can be found at ProbeBase^65^ and the sequences of the probes used are listed in Supplementary Table 6. The tissues were then incubated for 20 min with Alexafluor488-Phalloidin (130 nM) and 4,6-diamidino-2-phenylindole (1 μg ml^−1^) before mounting with Prolong reagent. The slides were imaged on a Cell Voyager CV100 using a × 40 oil immersion objective and stitched (90% overlap), and then analysed using Fiji imaging processing package.

### HEK-Blue hTLR4 and Null2 cell culture

HEK-Blue hTLR4 and Null2 cells (InvivoGen) were grown in complete growth medium (Dulbecco's modified Eagle's medium with 10% heat-inactivated fetal calf serum, 100 units per ml penicillin, 100 μg ml^−1^ streptomycin, 2 mM GlutaMAX, 1 mM pyruvate (Gibco, Life Technologies) and 100 μg ml^−1^ Normocin (InvivoGen)) in presence of cell line-specific selection antibiotics. Selection antibiotics for HEK-Blue hTLR4 cells included 100 μg ml^−1^ Zeocin, 200 μg ml^−1^ Hygrogold and 30 μg ml^−1^ Blasticidin, and for Null2 cells 100 μg /ml^−1^ Zeocin only (InvivoGen). The HEK-Blue hTLR4 reporter cell line was stably transfected with human TLR4, MD-2/CD14 co-receptor and the secreted embryonic alkaline phosphatase under the control of an nuclear factor kappa beta (NFκB)-responsive promoter. Null2 cells served as the parental control cell line and were stably transfected with the NFκB-responsive, secreted embryonic alkaline phosphatase reporter only. Cells were grown at 37 °C and 5% CO_2_.

### HEK-Blue TLR4 activation assay

*In vitro* stimulation of TLR4 activity by heat-inactivated bacteria was assessed in a similar method as previously described^66^. Briefly, bacteria were harvested, washed once in PBS−/− and resuspended in PBS−/− to an OD_600_ of 5, before being heat-inactivated at 80 °C for 1 h. Bacterial growth was tested to confirm efficient heat inactivation. HEK-Blue hTLR4 and Null2 cells (InvivoGen) were seeded at 50,000 cells in 100 μl per well of a 96-well plate and grown in complete growth medium as described above. Twenty-four hours after seeding, 100 μl of complete growth medium were added. Forty-eight hours after seeding, cells were washed once in complete growth medium without selection antibiotics and stimulated with 100 μl heat-inactivated bacteria in complete growth medium without selection antibiotics at an OD_600_ of 0.5. Twenty-four hours post stimulation, the supernatant was cleared by centrifugation at 740*g* for 10 min at room temperature and assayed for NFκB-responsive, secreted alkaline phosphatase activity using QUANTI-Blue reagent (InvivoGen). A_650_ was assessed at 37 °C. The averages of the cell-free medium and the Null2 cell controls were subtracted to obtain TLR4 activation of HEK-Blue hTLR4 cells.

### Statistical analysis

Statistical significance for the difference between two treatment groups was calculated by using a two-tailed Student's *t*-test or the Mann–Whitney *U*-test (for non-parametric data) unless otherwise stated. For assessing statistical significance among three or more groups, a one-way analysis of variance with *post hoc* Tukey's test was utilized. Statistical analysis was performed with assistance from GraphPad Prism Software Version 6.00 (GraphPad Software, San Diego California USA; www.graphpad.com). Statistical significance was given as ***P value <0.001, **P value <0.01 and *P value <0.05; NS (not significant) *P* value >0.05. The results are expressed as the mean value with s.e.m., unless otherwise indicated.

## Additional information

**How to cite this article:** Brown, E. M. *et al*. Diet and specific microbial exposure trigger features of environmental enteropathy in a novel murine model. *Nat. Commun.* 6:7806 doi: 10.1038/ncomms8806 (2015).

## Supplementary Material

Supplementary Information Supplementary Figures 1-14 and Supplementary Tables 1-6

Supplementary Data 1 An OTU table of the 454 pyrosequencing data derived from the 16S rDNA gene of small intestinal bacteria.

## References

[b1] Black R. E. . Maternal and child undernutrition: global and regional exposures and health consequences. Lancet 371, 243–260 (2008). 18207566 10.1016/S0140-6736(07)61690-0

[b2] Guerrant R. L., Oria R. B., Moore S. R., Oria M. O. & Lima A. A. Malnutrition as an enteric infectious disease with long-term effects on child development. Nutr. Rev. 66, 487–505 (2008). 18752473 10.1111/j.1753-4887.2008.00082.x PMC2562291

[b3] Kar B., Rao S. & Chandramouli B. A. Cognitive development in children with chronic protein energy malnutrition. Behav. Brain Funct. 4, 1–12 (2008). 18652660 10.1186/1744-9081-4-31 PMC2519065

[b4] Gordon J. I., Dewey K. G., Mills D. A. & Medzhitov R. M. The human gut microbiota and undernutrition. Sci. Transl. Med. 4, 137ps112 (2012). 10.1126/scitranslmed.3004347 22674549

[b5] Subramanian S. . Persistent gut microbiota immaturity in malnourished Bangladeshi children. Nature 510, 417–421 (2014). 24896187 10.1038/nature13421 PMC4189846

[b6] Yatsunenko T. . Human gut microbiome viewed across age and geography. Nature 486, 222–227 (2012). 22699611 10.1038/nature11053 PMC3376388

[b7] Shrimpton R. . Worldwide timing of growth faltering: implications for nutritional interventions. Pediatrics 107, e75–e75 (2001). 11331725 10.1542/peds.107.5.e75

[b8] Dewey K. G. & Adu-Afarwuah S. Systematic review of the efficacy and effectiveness of complementary feeding interventions in developing countries. Mat. Child Nutr. 4, 24–85 (2008). 10.1111/j.1740-8709.2007.00124.x PMC6860813 18289157

[b9] Trehan I. . Antibiotics as part of the management of severe acute malnutrition. New Engl. J. Med. 368, 425–435 (2013). 23363496 10.1056/NEJMoa1202851 PMC3654668

[b10] Korpe P. S. & Petri W. A. Jr. Environmental enteropathy: critical implications of a poorly understood condition. Trends Mol. Med. 18, 328–336 (2012). 22633998 10.1016/j.molmed.2012.04.007 PMC3372657

[b11] Prendergast A. & Kelly P. Enteropathies in the developing world: neglected effects on global health. Am. J. Trop. Med. Hyg. 86, 756–763 (2012). 22556071 10.4269/ajtmh.2012.11-0743 PMC3335677

[b12] Keusch G. T. . Environmental enteric dysfunction: pathogenesis, diagnosis, and clinical consequences. Clin. Infect. Dis. 59, S207–S212 (2014). 25305288 10.1093/cid/ciu485 PMC4481570

[b13] Petri W. A. Jr. & R N. C. a. H. Environmental enteropathy and malnutrition- do we know enough to intervene? BMC Med. 12, 187 (2014). 25604120 10.1186/s12916-014-0187-1 PMC4197320

[b14] Kosek M. . Assessment of Environmental Enteropathy in the MAL-ED Cohort Study: theoretical and analytic framework. Clin. Infect. Dis. 59, S239–S247 (2014). 25305293 10.1093/cid/ciu457 PMC4204611

[b15] Humphrey J. H. Child undernutrition, tropical enteropathy, toilets, and handwashing. Lancet 374, 1032–1035 (2009). 19766883 10.1016/S0140-6736(09)60950-8

[b16] Ahmed T. . An evolving perspective about the origins of childhood undernutrition and nutritional interventions that includes the gut microbiome. Ann. N. Y. Acad. Sci. 1332, 22–38 (2014). 25118072 10.1111/nyas.12487 PMC4514967

[b17] Ngure F. M. . Water, sanitation, and hygiene (WASH), environmental enteropathy, nutrition, and early child development: making the links. Ann. N. Y. Acad. Sci. 1308, 118–128 (2014). 24571214 10.1111/nyas.12330

[b18] Brown E. M., Arrieta M.-C. & Finlay B. B. A fresh look at the hygiene hypothesis: How intestinal microbial exposure drives immune effector responses in atopic disease. Sem. Immunol. 25, 378–387 (2013). 10.1016/j.smim.2013.09.003 24209708

[b19] Kau A. L., Ahern P. P., Griffin N. W., Goodman A. L. & Gordon J. I. Human nutrition, the gut microbiome and the immune system. Nature 474, 327–336 (2011). 21677749 10.1038/nature10213 PMC3298082

[b20] Simren M. . Intestinal microbiota in functional bowel disorders: a Rome foundation report. Gut 62, 159–176 (2013). 22730468 10.1136/gutjnl-2012-302167 PMC3551212

[b21] Hashimoto T. . ACE2 links amino acid malnutrition to microbial ecology and intestinal inflammation. Nature 487, 477–481 (2012). 22837003 10.1038/nature11228 PMC7095315

[b22] Visser J., Rozing J., Sapone A., Lammers K. & Fasano A. Tight junctions, intestinal permeability, and autoimmunity: celiac disease and type 1 diabetes paradigms. Ann. N. Y. Acad. Sci. 1165, 195–205 (2009). 19538307 10.1111/j.1749-6632.2009.04037.x PMC2886850

[b23] Rosenthal R. . Claudin-2, a component of the tight junction, forms a paracellular water channel. J. Cell. Sci. 123, 1913–1921 (2010). 20460438 10.1242/jcs.060665

[b24] Gu S. . Bacterial community mapping of the mouse gastrointestinal tract. PLoS ONE 8, e74957 (2013). 24116019 10.1371/journal.pone.0074957 PMC3792069

[b25] Assa A. . Vitamin D deficiency promotes epithelial barrier dysfunction and intestinal inflammation. J. Infect. Dis. 210, 1296–1305 (2014). 24755435 10.1093/infdis/jiu235

[b26] Schmidt D. R. . Regulation of bile acid synthesis by fat-soluble vitamins A and D. J. Biol. Chem. 285, 14486–14494 (2010). 20233723 10.1074/jbc.M110.116004 PMC2863217

[b27] Fasano A. Intestinal permeability and its regulation by zonulin: diagnostic and therapeutic implications. Clin. Gastroenterol. Hepatol. 10, 1096–1100 (2012). 22902773 10.1016/j.cgh.2012.08.012 PMC3458511

[b28] Fasano A. Zonulin, regulation of tight junctions, and autoimmune diseases. Ann. N. Y. Acad. Sci. 1258, 25–33 (2012). 22731712 10.1111/j.1749-6632.2012.06538.x PMC3384703

[b29] Konikoff M. R. & Denson L. A. Role of fecal calprotectin as a biomarker of intestinal inflammation in inflammatory bowel disease. Inflamm. Bowel Dis. 12, 524–534 (2006). 16775498 10.1097/00054725-200606000-00013

[b30] Coburn B., Grassl G. A. & Finlay B. Salmonella, the host and disease: a brief review. Immunol. Cell Biol. 85, 112–118 (2006). 17146467 10.1038/sj.icb.7100007

[b31] Feasey N. A., Dougan G., Kingsley R. A., Heyderman R. S. & Gordon M. A. Invasive non-typhoidal salmonella disease: an emerging and neglected tropical disease in Africa. Lancet 379, 2489–2499 (2012). 22587967 10.1016/S0140-6736(11)61752-2 PMC3402672

[b32] Syer S. D. & Wallace J. L. Environmental and NSAID-enteropathy: dysbiosis as a common factor. Curr. Gastroenterol. Rep. 16, 377 (2014). 24532193 10.1007/s11894-014-0377-1

[b33] Lin A. . Household environmental conditions are associated with enteropathy and impaired growth in rural Bangladesh. Am. J. Trop. Med. Hyg. 89, 130–137 (2013). 23629931 10.4269/ajtmh.12-0629 PMC3748469

[b34] Scharf R. J., Deboer M. D. & Guerrant R. L. Recent advances in understanding the long-term sequelae of childhood infectious diarrhea. Curr. Infect. Dis. Rep. 16, 408 (2014). 24819871 10.1007/s11908-014-0408-y PMC4157332

[b35] dos Reis J. C., de Morais M. B., Oliva C. A. & Fagundes-Neto U. Breath hydrogen test in the diagnosis of environmental enteropathy in children living in an urban slum. Digest. Dis. Sci. 52, 1253–1258 (2007). 17372830 10.1007/s10620-006-9288-9

[b36] Trehan I., Shulman R. J., Ou C. N., Maleta K. & Manary M. J. A randomized, double-blind, placebo-controlled trial of rifaximin, a nonabsorbable antibiotic, in the treatment of tropical enteropathy. Am. J. Gastroenterol. 104, 2326–2333 (2009). 19491826 10.1038/ajg.2009.270 PMC2758482

[b37] Mello C. S. . Methane production and small intestinal bacterial overgrowth in children living in a slum. World J. Gastroenterol. 18, 5932–5939 (2012). 23139610 10.3748/wjg.v18.i41.5932 PMC3491601

[b38] Ghoshal U. C. . Tropical sprue is associated with contamination of small bowel with aerobic bacteria and reversible prolongation of orocecal transit time. J. Gastroen. Hepatol. 18, 540–547 (2003). 10.1046/j.1440-1746.2003.03006.x 12702046

[b39] Swidsinski A., Weber J., Loening-Baucke V., Hale L. P. & Lochs H. Spatial organization and composition of the mucosal flora in patients with inflammatory bowel disease. J. Clin. Microbiol. 43, 3380–3389 (2005). 16000463 10.1128/JCM.43.7.3380-3389.2005 PMC1169142

[b40] Mukhopadhya I., Hansen R., El-Omar E. M. & Hold G. L. IBD—what role do Proteobacteria play? Nat. Rev. Gastroenterol. 9, 219–230 (2012). 10.1038/nrgastro.2012.14 22349170

[b41] Carvalho F. A. . Transient inability to manage proteobacteria promotes chronic gut inflammation in TLR5-deficient mice. Cell Host Microbe 12, 139–152 (2012). 22863420 10.1016/j.chom.2012.07.004 PMC4310462

[b42] Kau A. L. . Functional characterization of IgA-targeted bacterial taxa from undernourished Malawian children that produce diet-dependent enteropathy. Sci. Transl. Med. 7, 276ra224 (2015). 10.1126/scitranslmed.aaa4877 PMC4423598 25717097

[b43] Round J. L. & Mazmanian S. K. The gut microbiota shapes intestinal immune responses during health and disease. Nat. Rev. Immunol. 9, 313–323 (2009). 19343057 10.1038/nri2515 PMC4095778

[b44] Bloom S. M. . Commensal Bacteroides species induce colitis in host-genotype-specific fashion in a mouse model of inflammatory bowel disease. Cell Host Microbe 9, 390–403 (2011). 21575910 10.1016/j.chom.2011.04.009 PMC3241010

[b45] Wexler H. M. Bacteroides: the good, the bad, and the nitty-gritty. Clin. Microbial. Rev. 20, 593–621 (2007). 10.1128/CMR.00008-07 PMC2176045 17934076

[b46] Ramanan D. . Bacterial sensor Nod2 prevents inflammation of the small intestine by restricting the expansion of the commensal Bacteroides vulgatus. Immunity 41, 311–324 (2014). 25088769 10.1016/j.immuni.2014.06.015 PMC4238935

[b47] de Aguiar Vallim T. Q., Tarling E. J. & Edwards P. A. Pleiotropic roles of bile acids in metabolism. Cell Metab. 17, 657–669 (2013). 23602448 10.1016/j.cmet.2013.03.013 PMC3654004

[b48] Setchell K. D. R. . Genetic defects in bile acid conjugation cause fat-soluble vitamin deficiency. Gastroenteroly 144, 945–955 (2013). 10.1053/j.gastro.2013.02.004 PMC4175397 23415802

[b49] Hofmann A. F. & Eckmann L. How bile acids confer gut mucosal protection against bacteria. Proc. Natl Acad. Sci. USA 103, 4333–4334 (2006). 16537368 10.1073/pnas.0600780103 PMC1450168

[b50] Duboc H. . Connecting dysbiosis, bile-acid dysmetabolism and gut inflammation in inflammatory bowel diseases. Gut 62, 531–539 (2013). 22993202 10.1136/gutjnl-2012-302578

[b51] Zhang Y. G., Wu S., Xia Y. & Sun J. Salmonella infection upregulates the leaky protein claudin-2 in intestinal epithelial cells. PLoS ONE 8, e58606 (2013). 23505542 10.1371/journal.pone.0058606 PMC3594366

[b52] Calder P. C. Feeding the immune system. P. Nutr. Soc. 72, 299–309 (2013). 10.1017/S0029665113001286 23688939

[b53] Campbell D. I. . Chronic T cell-mediated enteropathy in rural west African children: relationship with nutritional status and small bowel function. Pediatr. Res. 54, 306–311 (2003). 12788978 10.1203/01.PDR.0000076666.16021.5E

[b54] Green P. H. R. & Cellier C. Celiac Disease. New Engl. J. Med. 357, 1731–1743 (2007). 17960014 10.1056/NEJMra071600

[b55] Schippa S. . A distinctive 'microbial signature' in celiac pediatric patients. BMC Microbiol. 10, 175 (2010). 20565734 10.1186/1471-2180-10-175 PMC2906462

[b56] Teodósio N., Lago E., Romani S. & Guedes R. A regional basic diet from northeast Brazil as a dietary model of experimental malnutrition. Arch Latinoam. Nutr. 40, 533–547 (1990). 2136514

[b57] Ueno P. M. . Alanyl-glutamine promotes intestinal epithelial cell homeostasis in vitro and in a murine model of weanling undernutrition. Am. J. Physiol. Gastrointest. Liver Physiol. 301, G612–G622 (2011). 21799183 10.1152/ajpgi.00531.2010 PMC3191556

[b58] Maier E. A. . Protein-energy malnutrition alters IgA responses to rotavirus vaccination and infection but does not impair vaccine efficacy in mice. Vaccine 32, 48–53 (2013). 24200975 10.1016/j.vaccine.2013.10.072 PMC3887447

[b59] Schloss P. D., Gevers D. & Westcott S. L. Reducing the effects of PCR amplification and sequencing artifacts on 16S rRNA-based studies. PLoS ONE 6, e27310 (2011). 22194782 10.1371/journal.pone.0027310 PMC3237409

[b60] Tautenhahn R., Bottcher C. & Neumann S. Highly sensitive feature detection for high resolution LC/MS. BMC Bioinformatics 9, 504 (2008). 19040729 10.1186/1471-2105-9-504 PMC2639432

[b61] Xia J., Mandal R., Sinelnikov I. V., Broadhurst D. & Wishart D. S. MetaboAnalyst 2.0—a comprehensive server for metabolomic data analysis. Nucleic Acids Res. 40, W127–W133 (2012). 22553367 10.1093/nar/gks374 PMC3394314

[b62] Han J. . Metabolic profiling of bile acids in human and mouse blood by LC–MS/MS in combination with phospholipid-depletion solid-phase extraction. Anal. Chem. 87, 1127–1136 (2015). 25496250 10.1021/ac503816u

[b63] Campbell J. M., Fahey G. C. & Wolf B. W. Selected indigestible oligosaccharides affect large bowel mass, cecal and fecal short-chain fatty acids, pH and microflora in rats. J. Nutr. 127, 130–136 (1997). 9040556 10.1093/jn/127.1.130

[b64] Hoiseth S. K. & Stocker B. A. Aromatic-dependent Salmonella typhimurium are non-virulent and effective as live vaccines. Nature 291, 238–239 (1981). 7015147 10.1038/291238a0

[b65] Loy A., Maixner F., Wagner M. & Horn M. probeBase—an online resource for rRNA-targeted oligonucleotide probes: new features 2007. Nucleic Acids Res. 35, D800–D804 (2007). 17099228 10.1093/nar/gkl856 PMC1669758

[b66] Shah N. R. . Minor modifications to the phosphate groups and the C3′ acyl chain length of lipid A in two Bordetella pertussis strains, BP338 and 18-323, independently affect toll-like receptor 4 protein activation. J. Biol. Chem. 288, 11751–11760 (2013). 23467413 10.1074/jbc.M112.434365 PMC3636864

