# Supplementary material for: Diet and specific microbial exposure trigger features of environmental enteropathy in a novel murine model
Source: Nat Commun. 2015 Aug 4;6:7806. doi: 10.1038/ncomms8806 (PMC4532793; doi:10.1038/ncomms8806)
Supplement: Supplementary Information — Supplementary Figures 1-14 and Supplementary Tables 1-6 [file ncomms8806-s1.pdf]

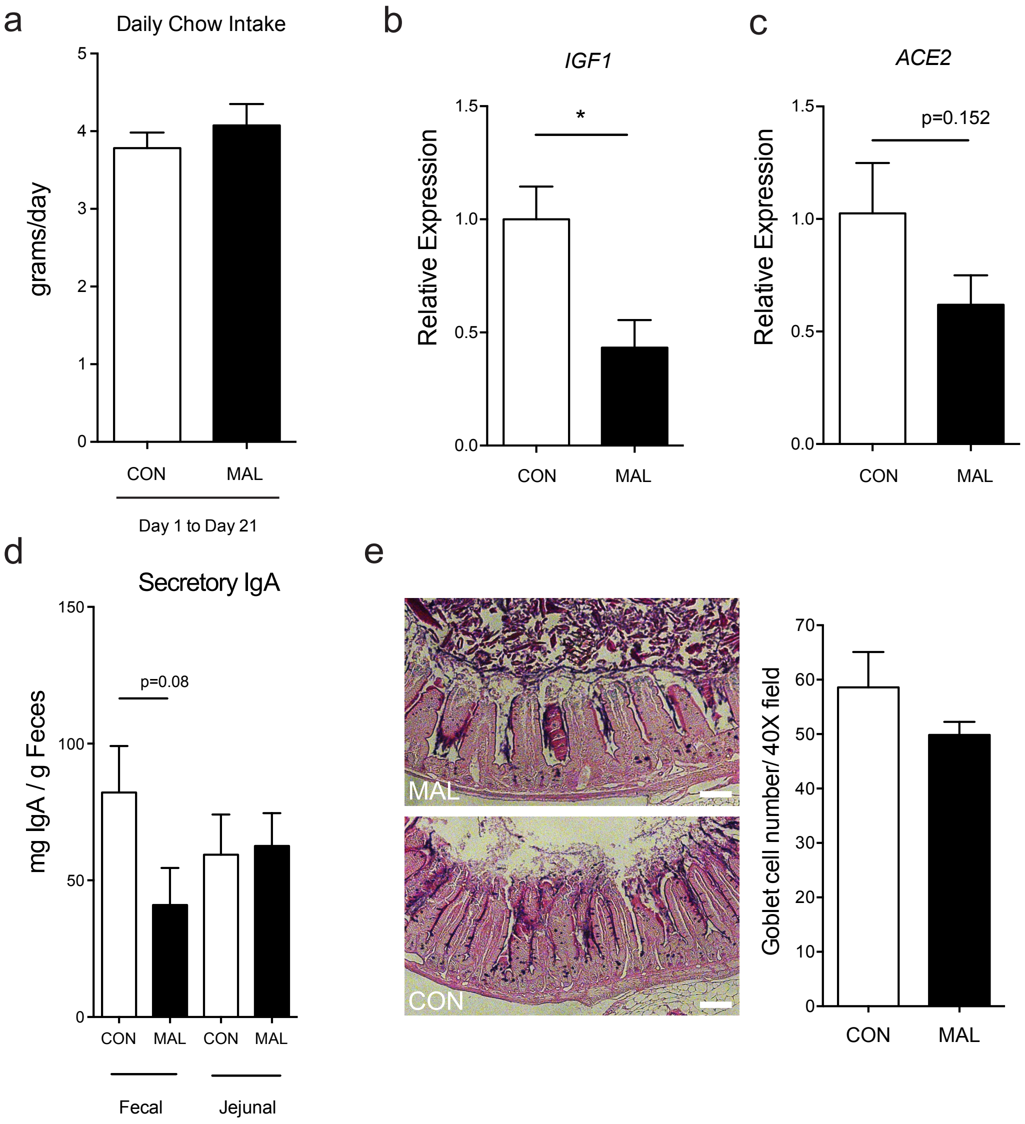

Supplementary Figure 1: Assessing gene expression, food intake and barrier function in malnourished and control mice. (**a**) The average daily intake of chow was determined by weighing the food each day and averaging difference in weight of the food over 3 weeks of mice on each of the diet. The mRNA expression of (**b**) *IGF1* and (**c**) *ACE2* in the jejunum was determined by real-time qPCR analysis. (**d**) The concentration of secretory IgA in the fecal content and jejunal content was assessed using an ELISA. (**e**) Representative images and histological assessment of AB-PAS stained jejunal tissues from malnourished and control mice. Mucins stain a blue or dark purple on the outer edge of each tissue, and mucus-secreting goblet cells stain dark purple in the epithelium. The number of goblet cells was enumerated and graphed for each tissue. Scale bar represents 100 μm in length. Bars indicate the mean with S.E, and all data are representative of 2 independent experiments, 8 mice per group (*p<0.05, Student's *t*-test).

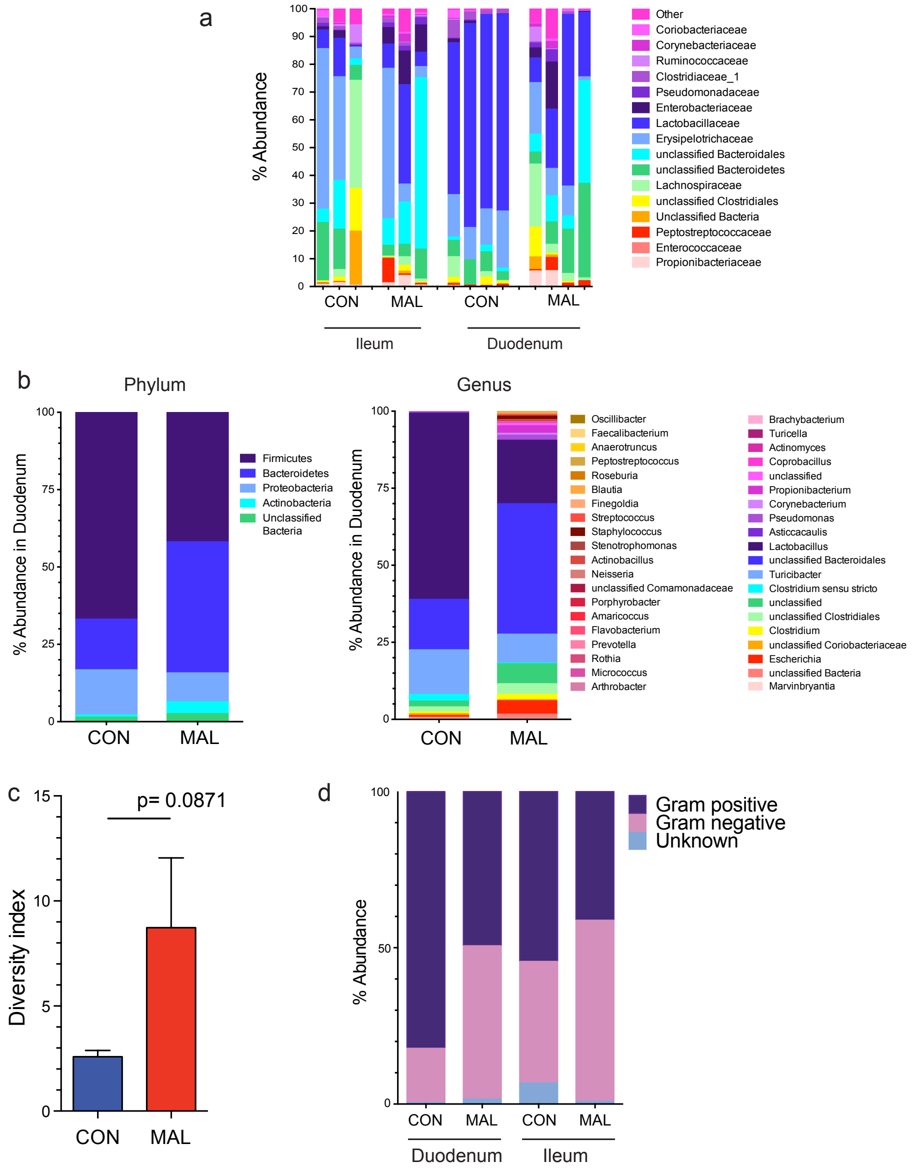

Supplementary Figure 2: High-throughput 16S rRNA sequencing of the small intestinal microbiota in malnourished and control mice. (**a**) A chart summarizing the percent abundance of the duodenal (n=4) and ileal (n=3) microbiota by family classification in each individual mouse, using the 16S rRNA gene. (**b**) The pooled percent abundance of the duodenal microbiota by phylum and genus taxonomic classification using the 16S rRNA gene. (**c**) A graph of the diversity in the duodenal microbiota of the malnourished and control mice (n=4) as measured by the inverse Simpson's Index method. Bars indicate the mean with S.E. (p=0.08, Student's *t*-test). (**d**) Percent abundance of OTUs that are either Gram-negative or Gram-positive in the duodenum and ileum of malnourished and control mice.

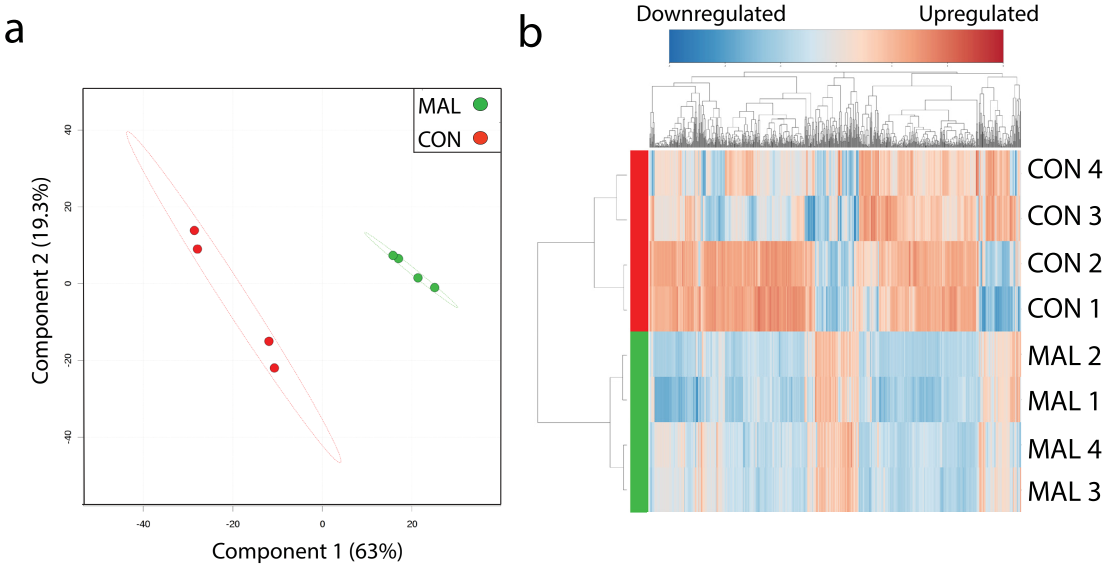

Supplementary Figure 3: Untargeted metabolomics of the small intestinal metabolome. (**a**) A PCA plot showing separation of the metabolomic data from the malnourished mice (green) and control mice (red) as detected by the negative ion channel. (**b**) A heat map of the relative abundance of all metabolites identified from the small intestinal metabolome as detected by the negative ion channel from malnourished and control mice (n=4). The malnourished and control samples clustered together in the dendogram based upon cluster analysis by the Ward method, with a Pearson distance measure. The heat map scale is a $\log_2$ base, from the range of -3 (blue) to +3 (red).

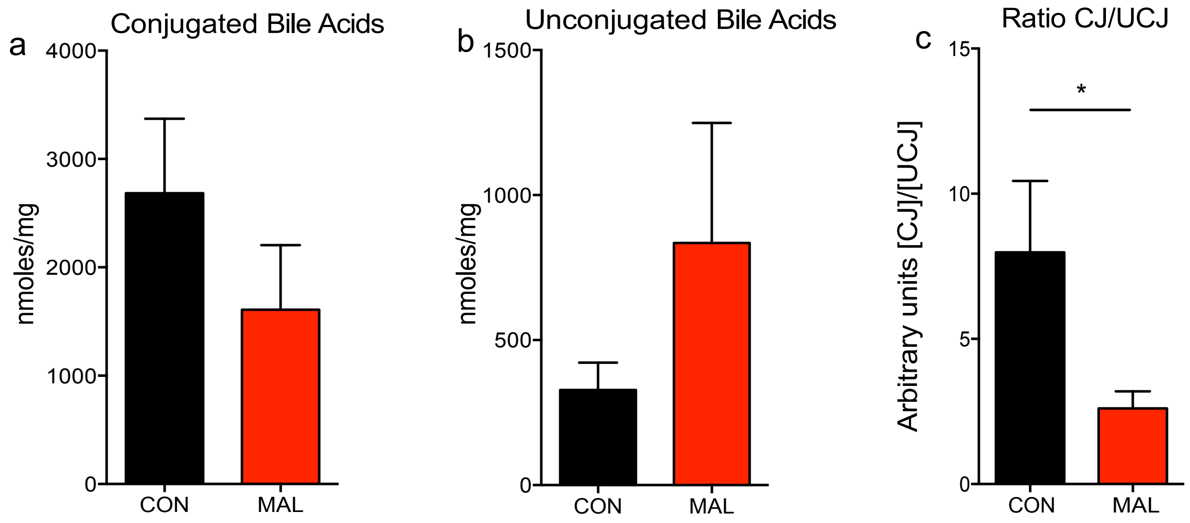

Supplementary Figure 4: Bile acid targeted metabolomics. Pooled data representing all bile acids found in the small intestinal content of malnourished and control mice that are either (**a**) tauro-conjugated or (**b**) unconjugated bile acids. (**c**) The ratio of the concentrations of conjugated:unconjugated bile acids in the small intestine. Bars indicate the mean with S.E, 3-4 mice per group (*$p<0.05$, Student's *t*-test).

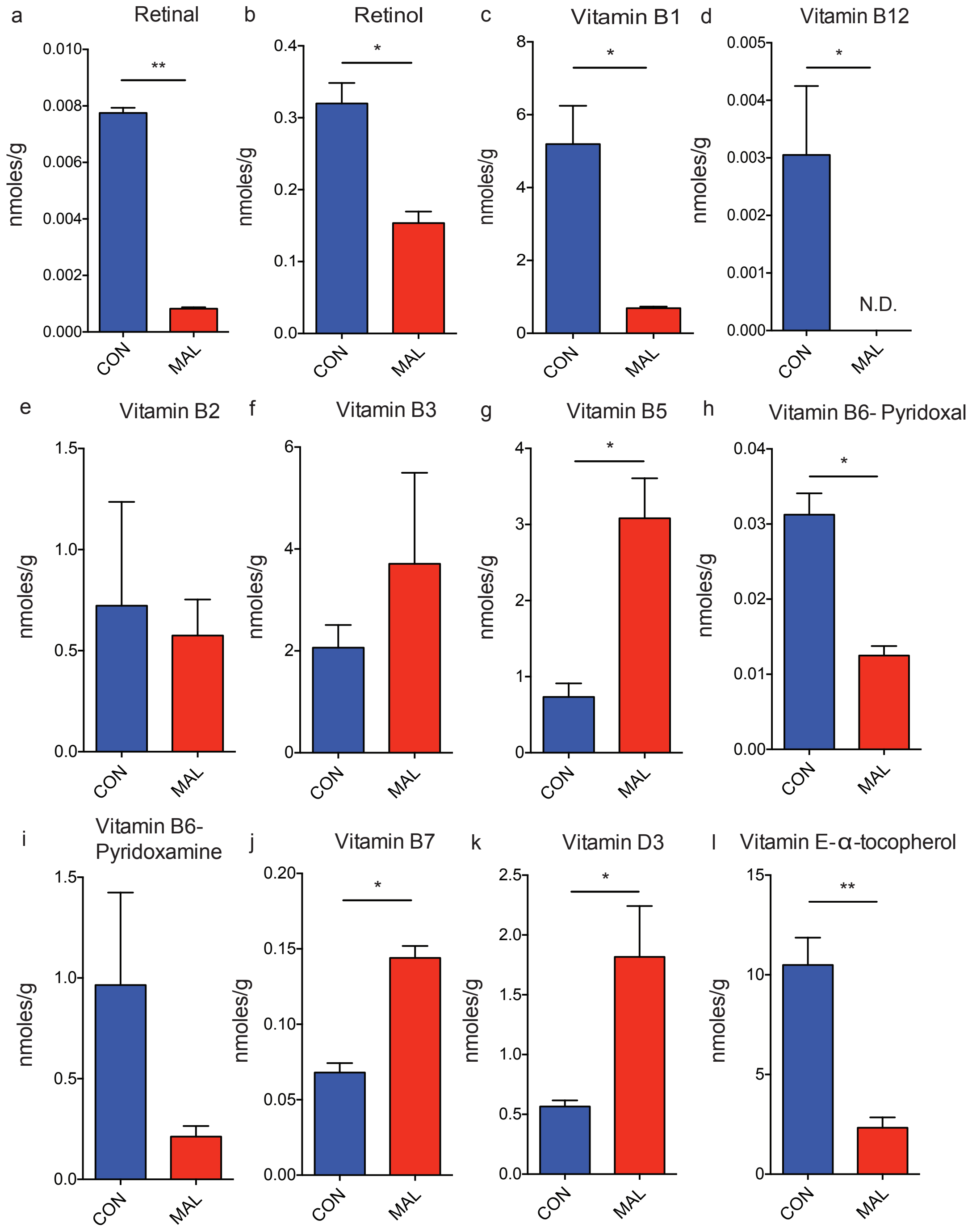

Supplementary Figure 5: Vitamin-targeted metabolomics. The concentrations of 12 vitamins found in the small intestinal content of malnourished and control mice. Bars indicate the mean with S.E, 4 mice per group, N.D. equals not detected. Statistical analysis was performed using the Mann-Whitney *U*-test (*p<0.05,**p<0.01)

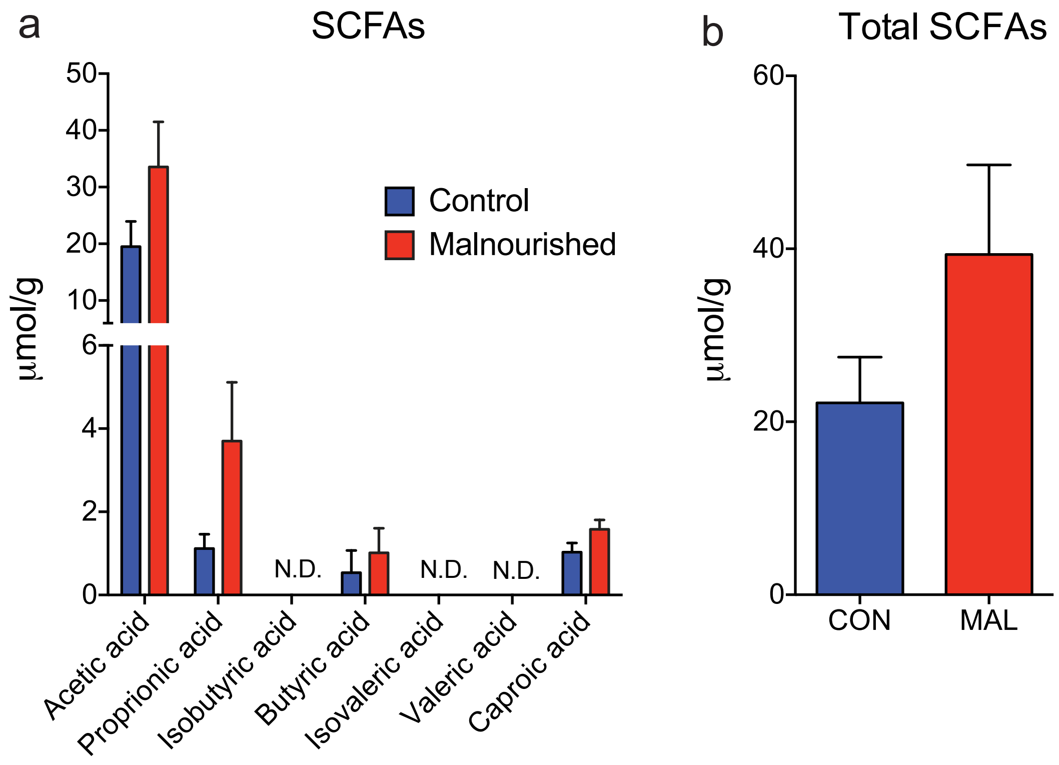

Supplementary Figure 6: Short-chain fatty acid analysis. (**a**) The concentration of 7 short-chain fatty acids (SCFAs) in the small intestinal content of malnourished and control mice. The concentrations of isobutyric, isovaleric and valeric acid were not detected (N.D.) in this analysis. (**b**) Pooled data representing the sum of all SCFAs found in the small intestine of malnourished and control mice in this analysis. Bars indicate the mean with S.E, 4 mice per group.

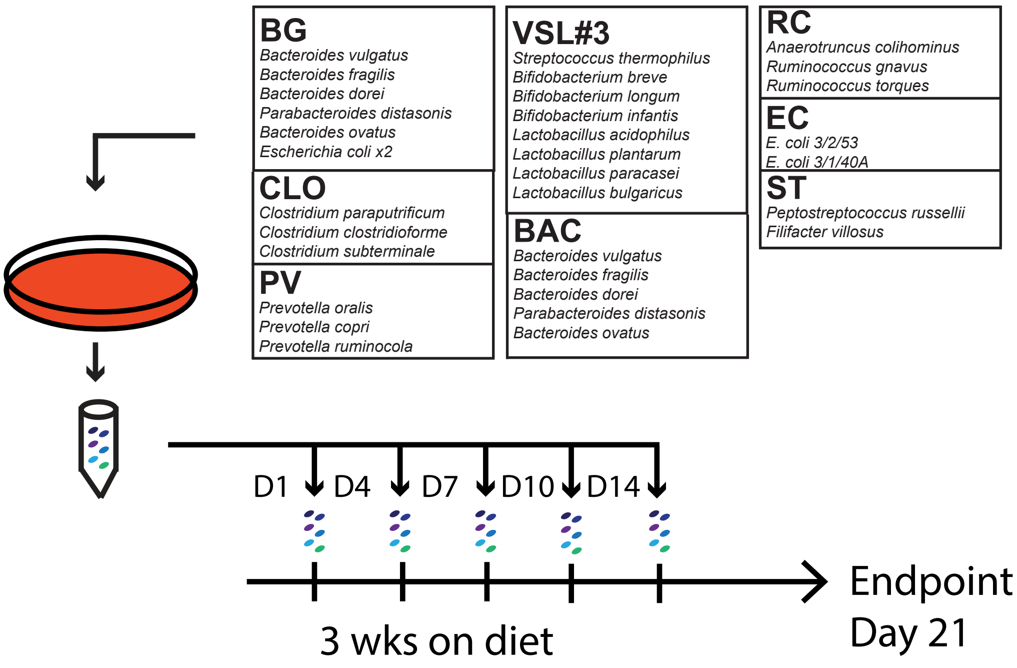

Supplementary Figure 7: Experimental design used to screen of the impact of oral exposure to various microbial mixtures on the growth rate, villous architecture and inflammatory markers in mice. A schematic of the experimental design used to administer the various microbial cocktails to malnourished mice. Three-week old mice were given the malnourished diet and exposed to each microbial mixture listed by oral gavage 5 times over a 2-week period. After the experimental endpoint 1 week later, mice were assessed for growth rate, tail-length, villous architecture and, in some cases, inflammation.

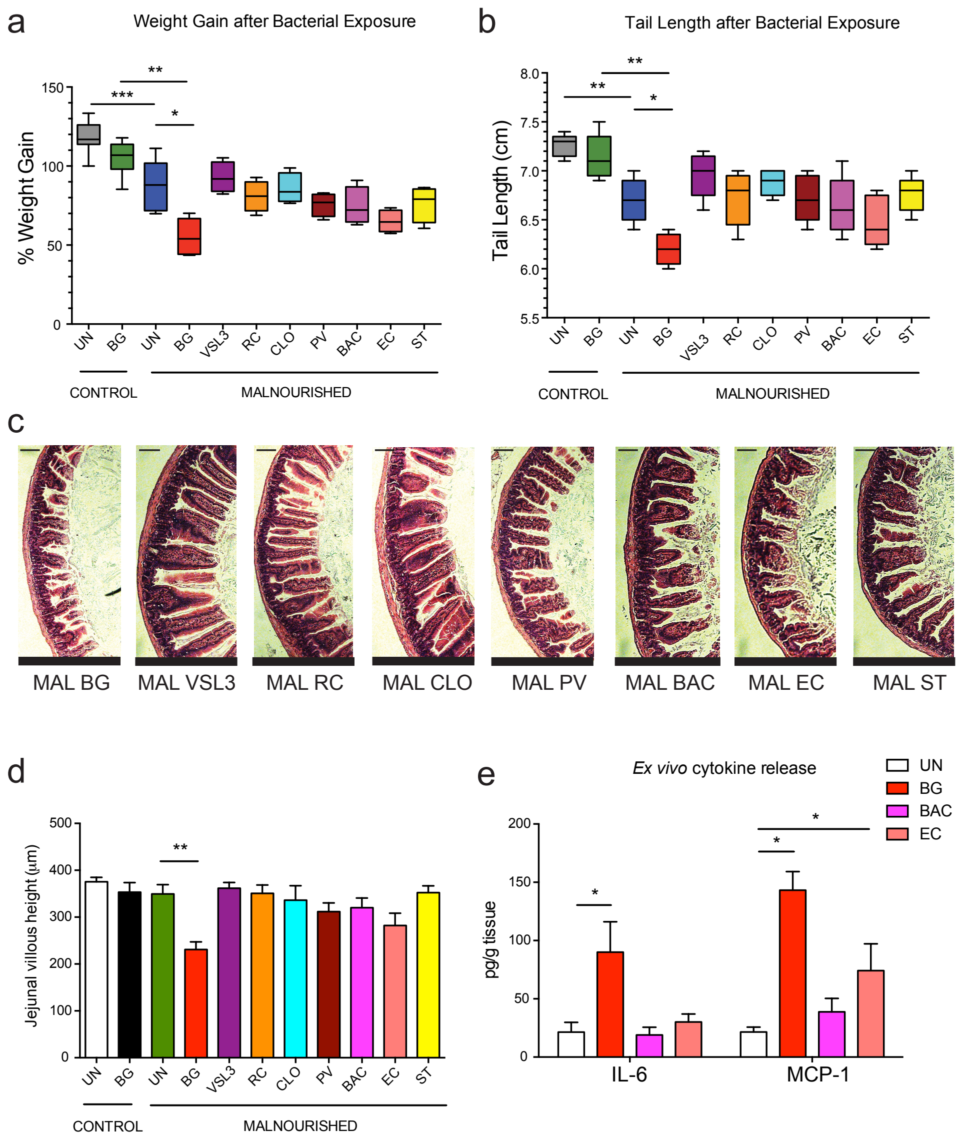

Supplementary Figure 8: A screen of the impact of oral exposure of C57Bl/6 mice to various microbial mixtures on growth rate, villous architecture and inflammatory markers in malnourished mice. After 3 weeks of exposure to each microbial mixture and being fed each diet, the total amount of (**a**) weight gained and (**b**) final tail lengths were calculated. Data on malnourished and control, unexposed mice (MAL-UN and CON-UN) are the same as presented in Figure 1, for comparison (n=8). The remaining data is based on 5 mice per group and is representative of 2 independent experiments (**c**) Representative images of the jejunal architecture from of H&E stained tissues from malnourished mice exposed to the microbial mixtures. Scale bar represents 100 μm in length (**d**) The histological assessment of villous height were measured in the malnourished and control mice exposed and unexposed to the microbial mixtures over a period of 3 weeks. Data from the images and graphs are representative of 2 independent experiments, 5 mice per group (**e**) Concentrations of IL-6 and MCP-1 released in the tissue culture media by jejunal sections of tissue from BG-exposed, EC-exposed, BAC-exposed and unexposed malnourished mice as measured by a cytokine bead array. Data is representative of 2 independent experiments, 5 mice per group. Statistical analysis was performed using a one-way ANOVA with post hoc Tukey's test (*p<0.05, **p,0.01, ***p<0.001).

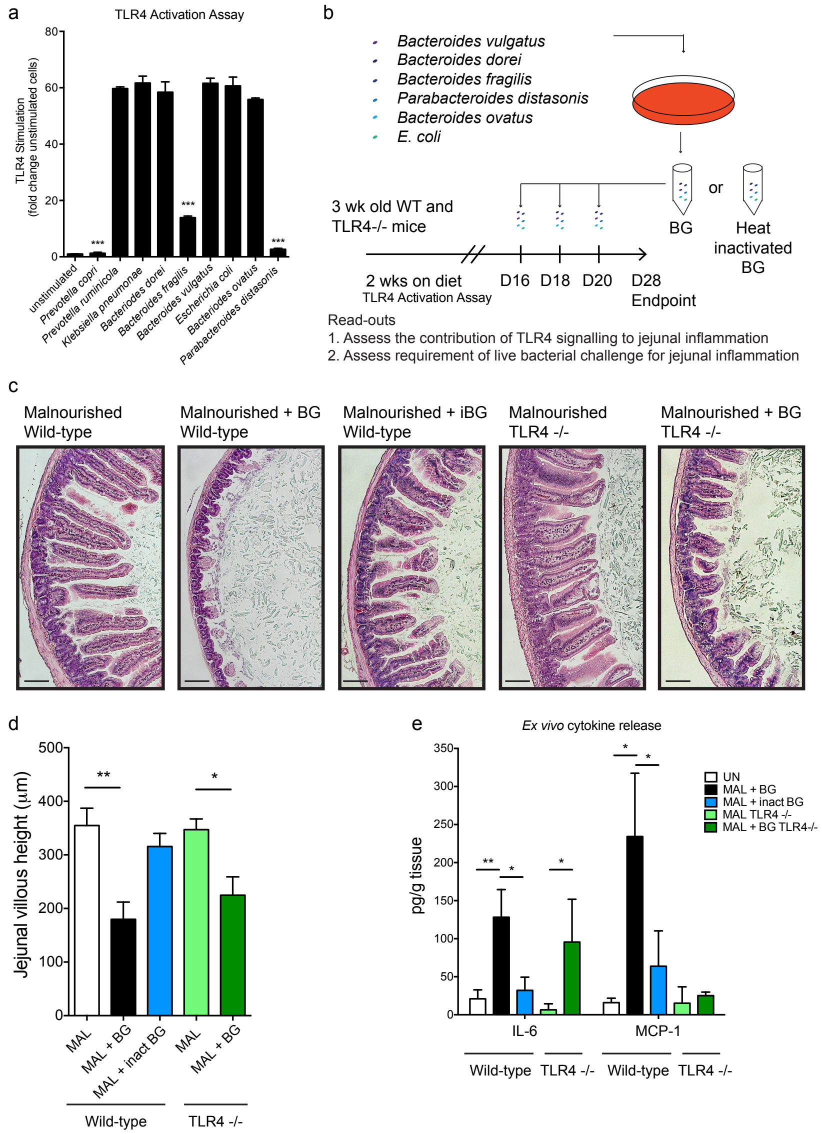

Supplementary Figure 9: An assessment of the requirement of TLR4 signaling and metabolically active bacteria in the inflammatory potential of the *Bacteroidales-E. coli* gavage. (**a**) A selection of Gram-negative LPS-containing microbes utilized in previous experiments (BG mix, *Prevotella* mix), along with a *Klebsiella* isolate were incubated with a TLR4 reporter cell line to assess the potential of each microbe to activate TLR4 signaling. Bars indicate the means, +/- S.E. of the fold change in activated compared to an unstimulated control. (**b**) A schematic of the experimental design for the following experiments in TLR4-deficient mice and wild-type mice given a heat-inactivated BG. (**c**) Representative images of the jejunal architecture from of H&E stained tissues in BG exposed and unexposed TLR4-deficient mice, wild type mice and wild-type mice given a heat-inactivated BG (iBG) mix. Scale bar represents 100 μm in length (**d**) Histological assessment of villous height in the malnourished and control mice exposed and unexposed to BG or heat-inactivated BG. Data from the images and graphs in wild-type mice are representative of 2 independent experiments, 8 mice per group. The TLR4-defecient mice data was based upon 6 mice per group. (**e**) Concentrations of IL-6 and MCP-1 released in the tissue culture media by jejunal sections of tissue from BG-exposed, inactivated BG exposed and unexposed malnourished mice as measured by a cytokine bead array. Bars indicate the means, +/- S.E. Statistical analysis was performed using a one-way ANOVA with post hoc Tukey's test (*p<0.05, **p,0.01).

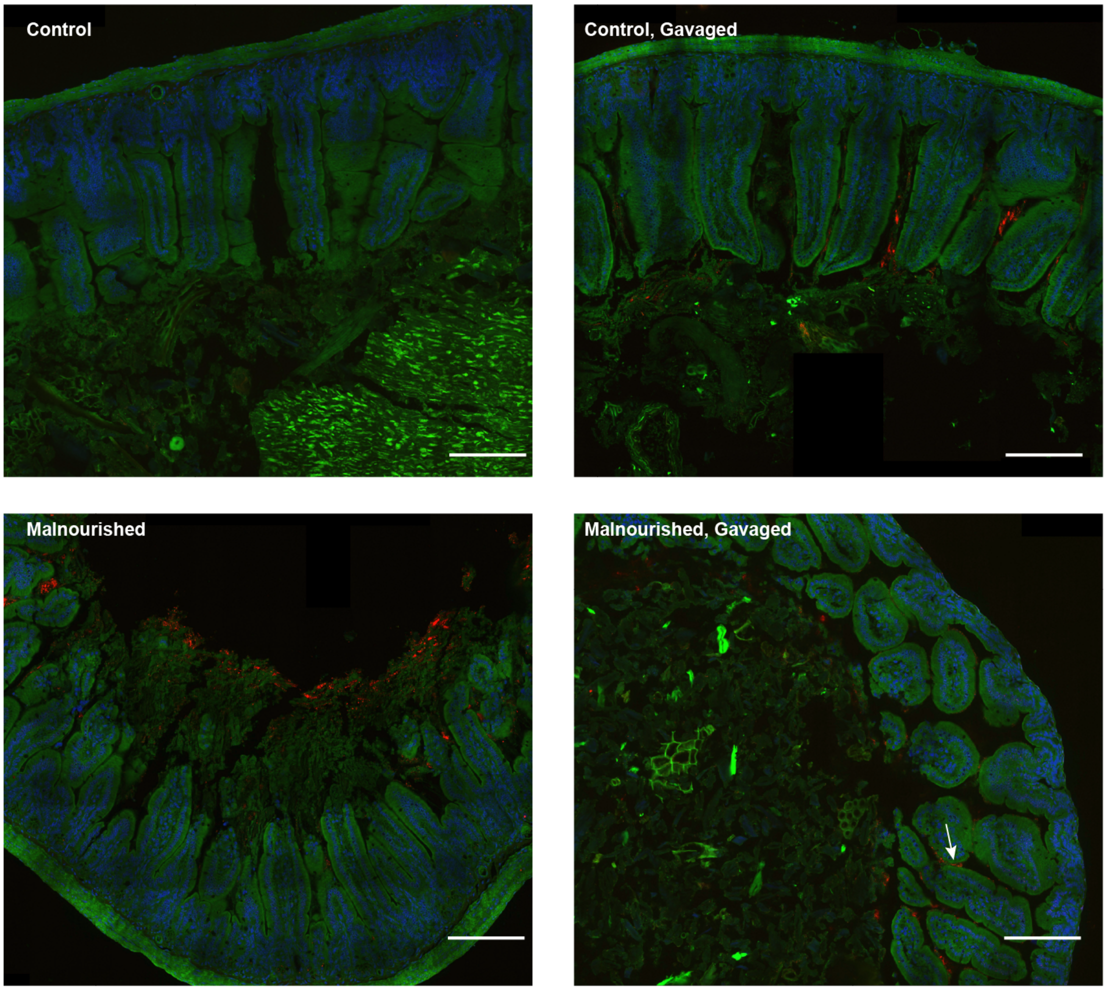

Supplementary Figure 10: FISH analysis of the Bacteroidetes in malnourished and control mice with or without *Bacteroidales-E. coli* oral exposure. Jejunal tissues preserved in Carnoy's solution were probed for Bacteroidetes-specific 16S rDNA (BAC) abundance using FISH. Images are representative of BG-exposed and unexposed malnourished and control mice. Actin is stained in green (488PHalloidin), cell nuclei in blue (DAPI) and bacteria are stained in red (Bac303). Scale bar indicates 100μm length, and arrows indicate tissue-associated Bacteroidetes.

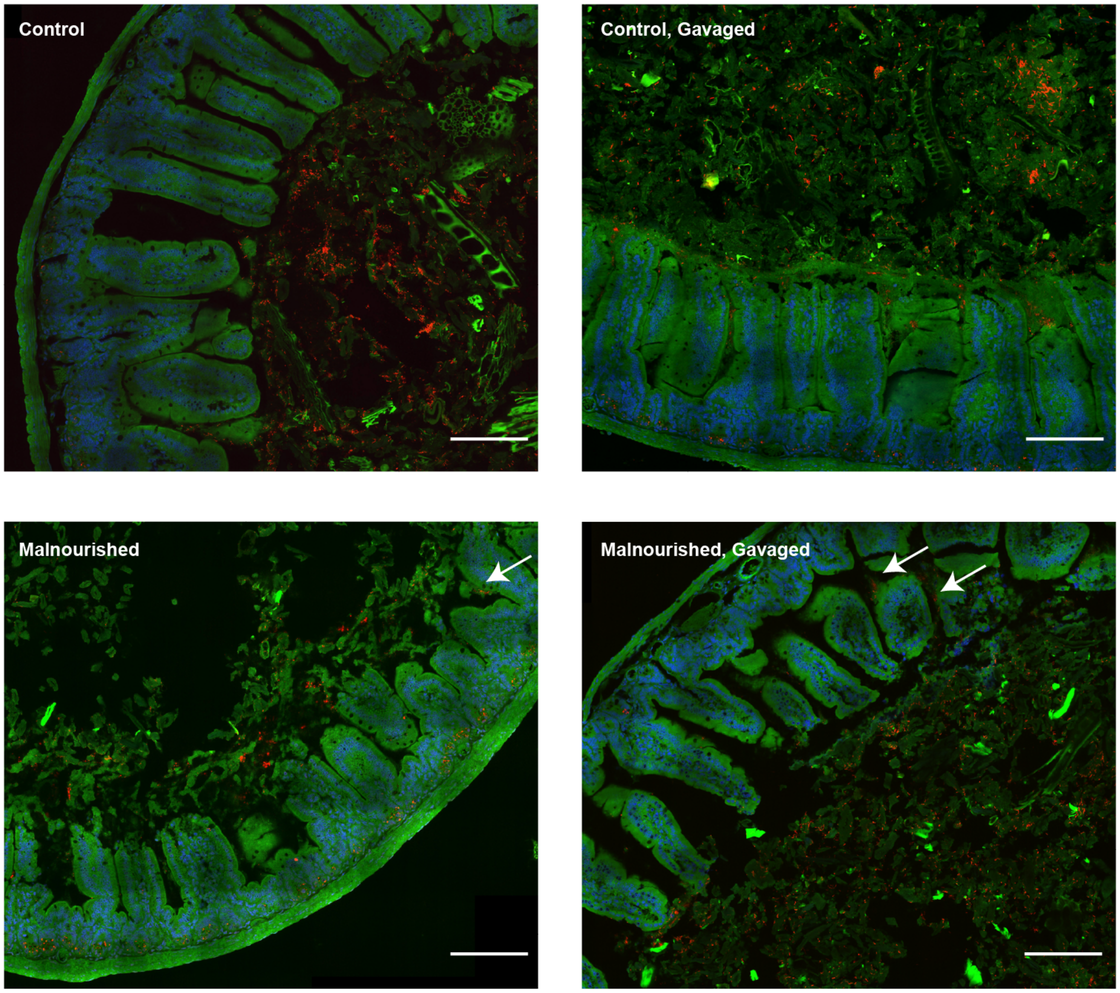

Supplementary Figure 11: FISH analysis of the Firmicutes in malnourished and control mice with or without *Bacteroidales-E. coli* oral exposure. Jejunal tissues preserved in Carnoy's solution were probed for Firmicutes-specific 16S rDNA abundance using FISH. Images are representative of BG-exposed and unexposed malnourished and control mice. Actin is stained in green (488PHalloidin), cell nuclei in blue (DAPI) and bacteria are stained in red (LGC354a-c). Scale bar indicates 100μm length, and arrows indicate tissue-assoicated Firmicutes.

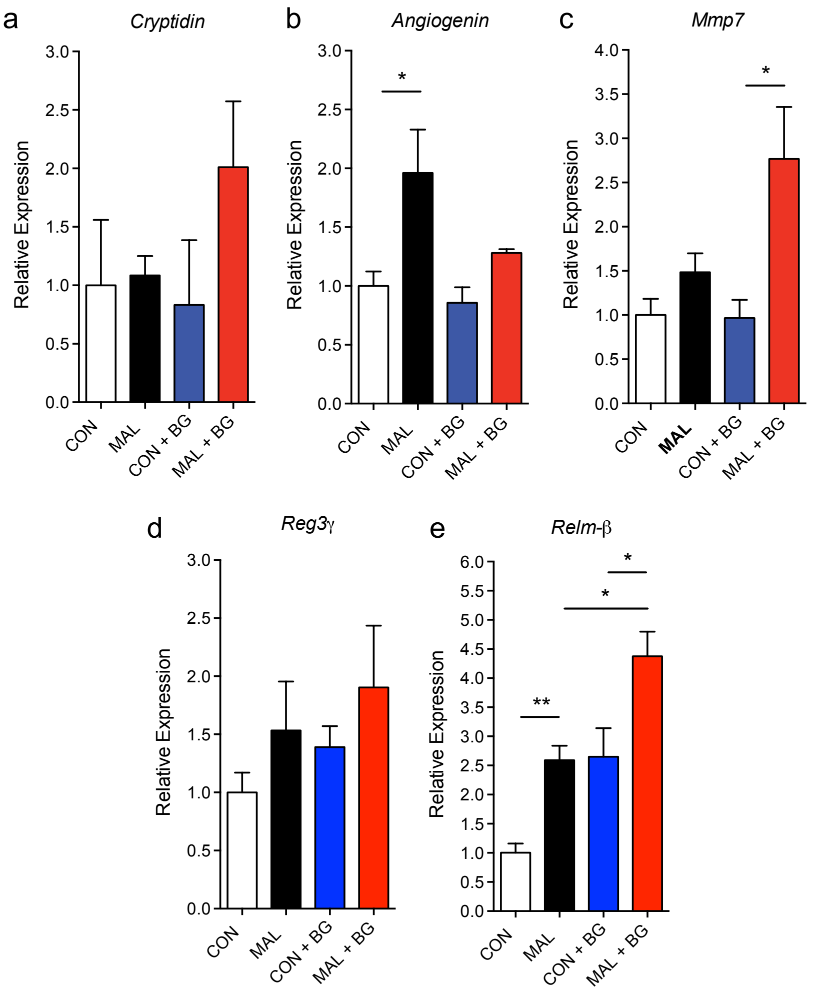

Supplementary Figure 12: Assessing the jejunal gene expression of antimicrobial defense proteins by RT-qPCR. The relative expression of (**a**) cryptidin, (**b**) angiogenin-4, (**c**) matrix metalloproteinase-7, (**d**) Reg3-γ and resistin-like molecule-β in the jejunum of BG exposed and unexposed mice on each diet was determined by real-time qPCR analysis. Graphs are representative of 2 independent experiments, 8 mice per group. Bars indicate the mean values +/- S.E. (*p<0.05, Student's *t*-test).

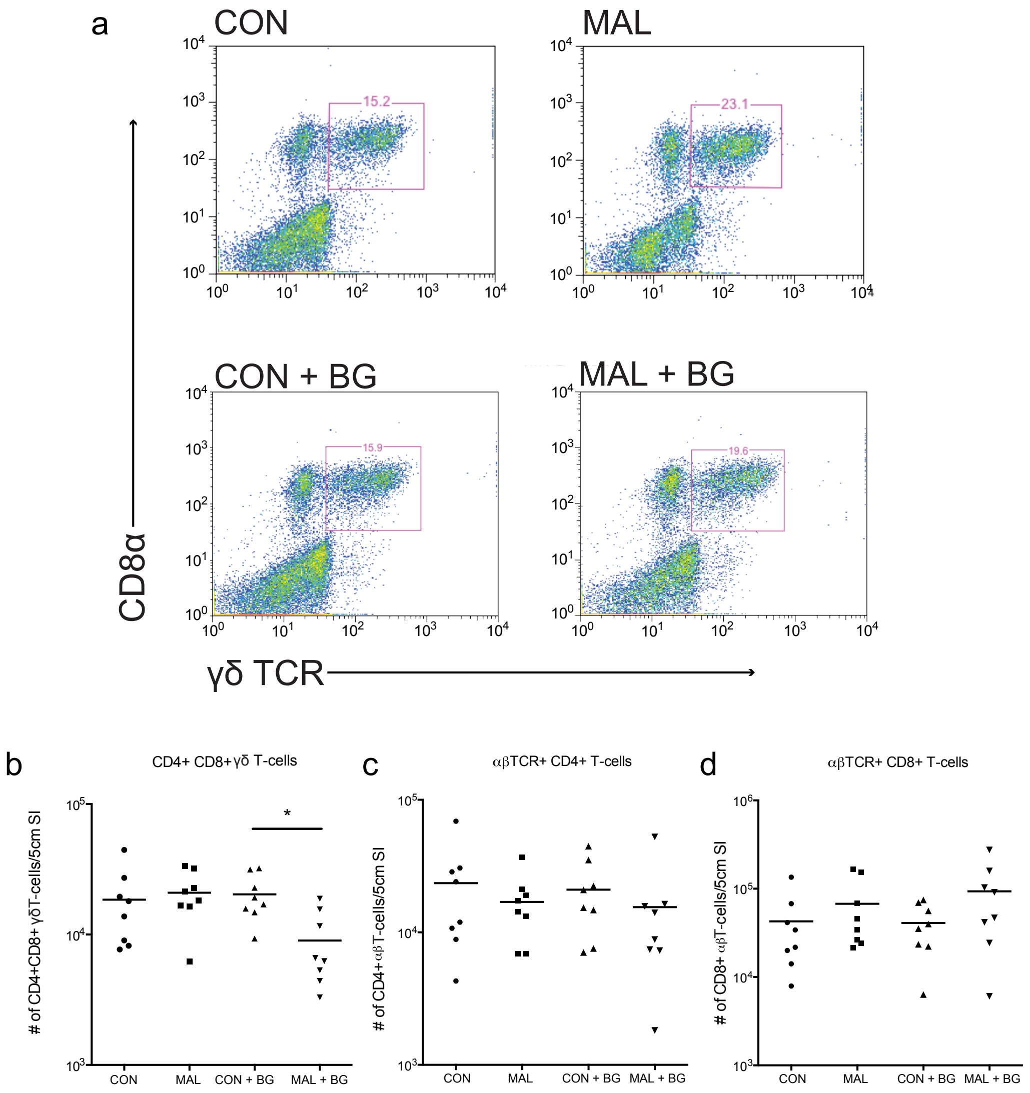

Supplemental Figure 13: Flow cytometry of small intestinal intraepithelial lymphocytes. (**a**) A graph generated by FlowJo showing the gating of CD8+γδTCR+ T-cells from CD45+ live lymphocytes isolated from the upper 5 cm of the small intestine (duodenum) in BG-exposed and unexposed mice on each diet. The total number of (**b**) CD45+CD3+CD8+CD4+γδTCR+ cells, (**c**) CD45+CD3+CD4+αβTCR+ cells and (**d**) CD45+CD3+CD8+αβTCR+ cells isolated from a 5 cm portion of the duodenum in BG-exposed and unexposed mice on each diet. All data are representative of 2 independent experiments (n=8). Bars indicate the mean values (*$p<0.05$, one-way ANOVA with post hoc Tukey's test).

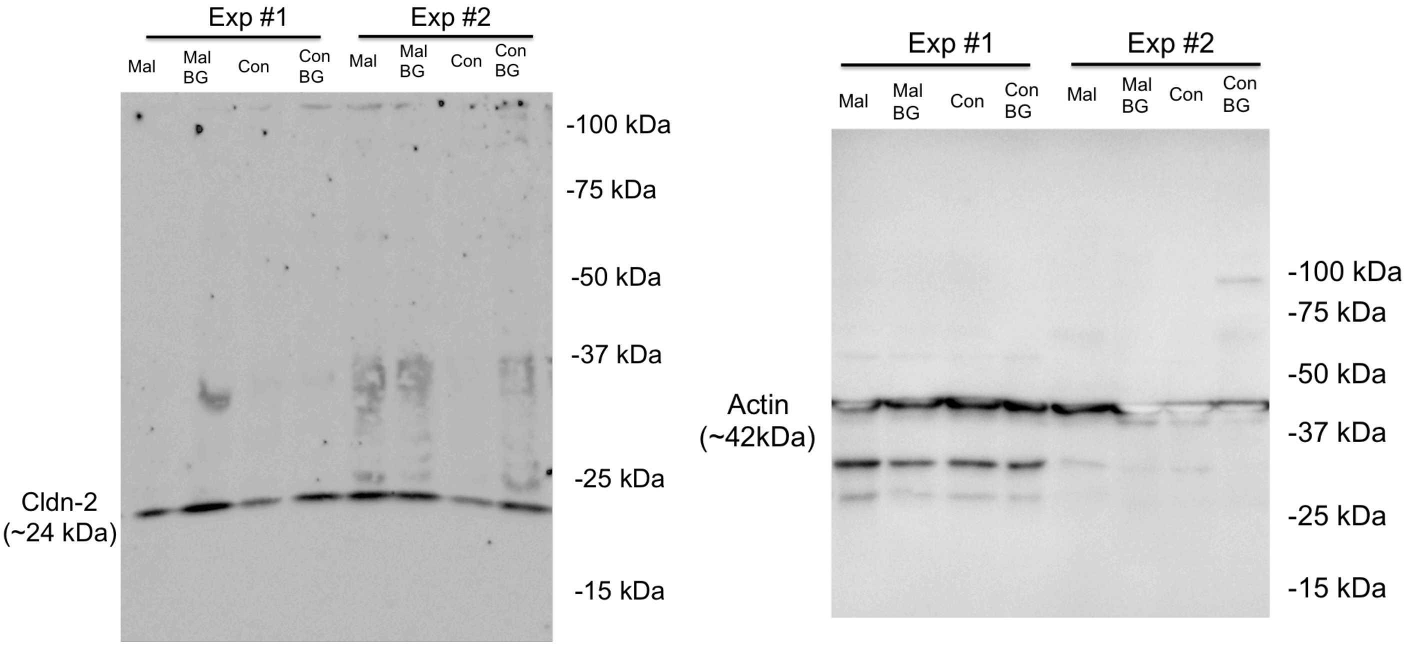

Supplementary Figure 14: Uncropped claudin-2 western blot from jejunal epithelial cells. Levels of CLDN2 protein blotted from extracted protein from jejunal IECs. Single bands can be visualized in the expected region for the protein claudin-2 (~24kDa). Actin was used as the loading control, and a consistent abundance of actin could be visualized across all samples.

Supplementary Table 1: A breakdown of the ingredients and calorie content in the malnourished and control diet.

	Control Diet		Malnourished Diet	
**Ingredients**	**Grams (%)**	**Kcal (%)**	**Grams (%)**	**Kcal (%)**
Casein	200	800	71	284
L-Cystine	3	12	1.07	4
Corn Starch	346	1384	557	2228
Maltodextrin 10	45	180	70	280
Dextrose	250	1000	250	1000
Sucrose	0	0	2.41	10
Cellulose BW200	75	0	75	0
Inulin	25	25	25	25
Soybean Oil	70	630	23.3	210
Mineral Mix S10026	10	0	10	0
Dicalcium Phosphate	13	0	13	0
Calcium Carbonate	5.5	0	5.5	0
Potassium Citrate, 1 H2O	16.5	0	16.5	0
Vitamin Mix V10001	10	40	10	40
Choline Bitartrate	2	0	2	0
Red Dye #40, FD&C	0	0	0.05	0
Blue Dye #1, FD&C	0.025	0	0	0
Yellow Dye #5, FD&C	0.025	0	0	0
**Total**	**1071.05**	**4071**	**1131.83**	**4081**
Protein	19.0	20	6.4	7
Carbohydrates	63.1	65	80.6	88
Fat	6.5	15	2.1	5
**Total**		**100**		**100**
**kcal/gm**	**3.77**		**3.77**	

Supplementary Table 2: A list of the top 10 most significant OTUs in the duodenal microbiome in malnourished and control mice. Green indicates OTUs that were increased in malnourished mice and red indicates OTUs that were decreased in malnourished mice.

**OTU Classification**	**Rank**	**Relative Abundance (%)**		***P*-value***
		**Con**	**Mal**	
Escherichia_Shigella	Genus	0.056	5.21	0.2890
Unclassified Bacteroidales	Order	1.27	14.46	0.0286
Unclassified Bacteroidetes	Phylum	6.30	15.57	0.2000
Lachnospiraceae	Family	2.40	7.44	0.3429
Unclassified Clostridiales	Order	1.48	2.99	0.3838
Pseuodomonas	Genus	0.18	1.53	0.2486
Prevotella	Genus	0.00	0.062	0.1878
Peptostreptococcaceae	Family	0.00	0.21	0.1143
Ruminococcaceae	Family	0.08	1.44	0.3297
Lactobacillus	Genus	67.22	28.82	0.0571
Turicibacter	Genus	15.09	10.09	0.2603
Clostridiaceae	Family	3.06	0.36	0.0286

*Statistical analysis performed using the Mann-Whitney *U*-test.

Supplementary Table 3: Most significant metabolite features in the small intestine of malnourished (yellow) and control (blue) mice as determined by the Random Forest algorithm. Biochemical names are given as the closest match within 3 ppm of mz on the METLIN database.

Biochemical Name	MZ/RT	Mean Decrease Accuracy*	P-value	Fold Change	Pathway
25-hydroxyvitamin D3	401.34099/ 13.97	0.010197	0.0126	9.261121114	Vitamin D metabolism
5,9-hexacosadienoic acid	415.36048/ 10.32	0.010167	0.07584	9.984408818	Fatty acid metabolism
Not determined	480.27765/ 4.13	0.0095	0.06038	4.81442954	N/A
Dehydroepiandrosterone 3-glucuronide	487.23114/ 12.14	0.0091667	0.02146	4.377919983	Steroid biosynthesis
Not determined	557.36625/ 7.08	0.009	0.02101	9.837171854	N/A
Not determined	308.29477/ 8.99	0.009	0.05241	6.7656939	N/A
D-Urobilinogen	591.31751/ 6.39	0.009	0.16924	51.61731217	Bilirubin metabolism
Not determined	432.32318/ 13.48	0.0085667	0.04659	4.991777729	N/A
L-tyrosine	182.08116/ 0.52	0.0085	0.05542	4.490194451	Amino acid metabolism
7-ketodeoxycholic acid	407.27953/ 6.07	0.0081667	0.01052	5.878640664	Bile acid metabolism
Not determined	555.42304/ 13.37	0.0081667	0.00057	4.958823421	N/A
Not determined	426.30034/ 12.46	0.0081667	0.10915	12.26049061	N/A
Not determined	484.33006/ 9.13	0.008	0.08897	6.034367899	N/A
Phytenoic Acid	311.29444/ 13.82	0.0078333	0.07204	4.807276589	Fatty acid metabolism
Not determined	574.39284/ 7.07	0.0076667	0.01903	7.444808495	N/A
Not determined	376.27345/ 13.63	0.0075667	2.67E-06	5.583238881	N/A
LysoPC(20:0)	574.38654/ 11.4	0.0075	0.00184	3.835402686	Lipid metabolism
N-oleoyl tyrosine	446.32632/ 12.64	0.0075	0.10891	7.527675391	Fatty acid metabolism
Dihydroxycholestanoic acid	435.35087/ 7.49	0.0075	0.12485	7.282721622	Bile acid metabolism
Not determined	479.25137/ 13.01	0.0075	0.00873	4.695383928	N/A
Not determined	454.33135/ 13.95	0.0075	0.09990	7.979301	N/A
4α-formyl-4β-methyl-5α-cholesta-8,24-dien-3β-ol	443.35179/ 13.94	0.0074667	0.00077	26.05893193	Cholesterol metabolism
Not determined	466.2619/3. 5	0.0073333	0.03404	3.836387738	N/A
Not determined	418.34393/ 14.28	0.0073333	0.00970	4.915347782	N/A
Not determined	444.35515/ 13.95	0.0073333	0.00129	34.1154967	N/A

Not determined	406.26682/5.34	0.0073333	0.01889	3.72010044	N/A
Etioporphyrin III	501.29738/11.62	0.0072333	0.01934	9.073079574	Heme degradation
Not determined	457.21135/2.72	0.0072333	0.02592	57.5444817	N/A
Not determined	710.44126/11.56	0.0072333	0.00701	12.0588179	N/A
PC(18:3(9Z,12Z,15Z)/0:0)	518.32392/8.5	0.007	0.06521	13.32279068	Lipid metabolism
Not determined	307.18479/6.72	0.007	0.11179	34.19762002	N/A
Not determined	524.26499/3.51	0.007	0.04709	3.035796468	N/A
Not determined	836.537/6.12	0.007	0.00599	3.845719942	N/A
N-palmitoyl phenylalanine	404.31568/14.25	0.007	0.01364	5.259420743	Fatty acid metabolism
PECer(d15:2(4E,6E)/20:0(2OH))	689.52115/13	0.007	0.01912	10.07889011	Lipid metabolism
Not determined	553.40568/13.43	0.007	0.01399	3.883426116	N/A
Not determined	502.28322/3.5	0.0069667	0.05137	3.593148889	N/A
N-Oleoyl-D-erythro-sphingosine	550.5192/15.79	0.0068333	0.01158	5.202454741	Sphingolipid metabolism
Hexadecanedioic acid mono-L-carnitine ester	430.31611/5.92	0.0068333	0.00912	14.83229637	Lipid metabolism
N-cis-octadec-9Z-enoyl-L-Homoserine lactone	366.30009/8.03	0.0068333	0.03900	5.115979822	Quorum sensing

*sorted in order determined by the mean decrease accuracy.

Supplementary Table 4: A description of all human-derived commensal bacterial strains utilized in this study. Strains provided by E.A.V are in highlighted in blue, DSMZ in yellow, VSL#3 in purple, and ATCC in green.

Cocktail	Acronym	Strains	Source
Bacteroides-E. coli	BG	*Bacteroides vulgatus* 3/1/40A<br>*Bacteroides fragilis* 3/1/12<br>*Bacteroides ovatus* 3/8/47<br>*Bacteroides dorei* 5/1/36 (D4)<br>*Parabacteroides distasonis* 2/1/33B<br>*Escherichia coli* 3/2/53<br>*Escherichia coli* 4/1/47	Biopsies and Feces
VSL3 Probiotic Mix	VSL3	*Streptococcus thermophilus*<br>*Bifidobacterium breve*<br>*Bifidobacterium longum*<br>*Bifidobacterium infantis*<br>*Lactobacillus acidophilus*<br>*Lactobacillus plantarum*<br>*Lactobacillus paracasei*<br>*Lactobacillus bulgaricus*	VSL#3® (Sigma-tau pharmaceuticals Inc., Gaithersburg, MD)
Ruminococcus Mix	RC	*Anaerotruncus colihominus* DSM 17241<br>*Ruminococcus gnavus* 2/1/58<br>*Ruminococcus torques* 3/1/46	Feces
Clostridium Mix	CLO	*Clostridium paraputrificum*<br>*Clostridium clostridioforme*<br>*Clostridium subterminale*	Feces and biopsies
Prevotella Mix	PV	*Prevotella oralis* CC98A<br>*Prevotella copri* DSM 18205<br>*Prevotella ruminocola* ATCC 19189	Feces and biopsies
Bacteroides Mix	BAC	*Bacteroides vulgatus* 3/1/40A<br>*Bacteroides fragilis* 3/1/12<br>*Bacteroides ovatus* 3/8/47<br>*Bacteroides dorei* 5/1/36 (D4)<br>*Parabacteroides distasonis* 2/1/33B	Biopsies and Feces
Enterobacteriaceae Mix	EC	*E. coli* 3/2/53<br>*E. coli* 4/1/47	Biopsies
Peptostreptococcaceae Mix	ST	*Peptostreptococcus russellii* DSM 23041<br>*Filifacter villosus* DSM 1645	Feces

Supplementary Table 5: A list of all qPCR primers and sequences utilized in this study for host gene expression and assessment of bacterial 16S rDNA abundance.

**Host Gene Target**	**Sequence (5' -> 3')**	**Annealing Temp. (°C)**
TJP1	Fwd- CCCTGAAAGAAGCGATTCAG<br>Rev- CCCGCCTTCTGTATCTGTGT	60
CLDN2	Fwd- ATACTACCCTTTAGCCCTGACCGAGA<br>Rev- CAGTAGGAGCACACATAACAGCTACCAC	60
CLDN4	Fwd- CGCTACTCTTGCCATTACG<br>Rev- ACTCAGCACACCATGACTTG	60
CLDN15	Fwd- GCAGGGACCCTCCACATATTG<br>Rev- AGTTCATACTTGGTTCCAGCATACGTG	60
IGF1	Fwd- TTCAGTTCGTGTGTGGACCGAG<br>Rev- TCCACAATGCCTGTCTGAGGTG	60
ACE2	Fwd- TGGTCTTCTGCCATCCGATT<br>Rev- CCATCCACCTCCACTTCTCTAA	60
CRYP	Fwd- GAGAGATCTGGTATGCTATTG<br>Rev- AGCAGAGTGTGTACATTAAAT	60
ANG	Fwd- CTCTGGCTCAGAATGAAAGGTACGA<br>Rev- GAAATCTTTAAAGGCTCGGTACCC	60
REG3	Fwd- AAGCTTCCTTCCTGTCCTCC<br>Rev- TCCACCTCTGTTGGGTTCAT	60
MMP7	Fwd- CACTCTAGGTCATGCCTTCGC<br>Rev- GGTGGCAGCAAACAGGAAGTT	60
RELMB	Fwd- GCTCTTCCCTTTCCTTCTCCAA<br>Rev- AACACAGTGTAGGCTTCATGCTGTA	60
GAPDH	Fwd- ATTGTCAGCAATGCATCCTG<br>Rev- ATGGACTGTGGTCATGAGCC	60
**Bacterial Target**		
Eubacteria 16S rRNA (total bacteria)	Fwd- ACTCCTACGGGAGGCAGCAGT<br>Rev- ATTACCGCGGCTGCTGGC	60
Bacteroidetes 16S rRNA	Fwd- GGTTCTGAGAGGAAGGTCCC<br>Rev- GCTGCCTCCCGTAGGAGT	60

Clostridium cluster IV	Fwd- ACAATAAGTAATCCACCTGG<br>Rev- CTTCCTCCGTTTTGTCAA	60
Clostridium cluster XIVa	Fwd- ACTCCTACGGGAGGCAGC<br>Rev- GCTTCTTAGTCAGGTACCGTCAT	60
Lactobacillus/Lactococcus	Fwd- AGCAGTAGGGAATCTTCCA<br>Rev- CACCGCTACACATGGAG	60
Enterobacteriaceae 16S rRNA	Fwd-CATTGACGTTACCCGCAGAAGAAGC<br>Rev- CTCTACGAGACTCAAGCTTGC	56

Supplementary Table 6: A list of all FISH probes utilized in this study, along with the sequence and formamide concentrations used.

Probe Name	Target	Sequence (5’ -> 3’)	Formamide Concentration
Eub338	Total Bacteria (Eubacteria)	5'- GCT GCC TCC CGT AGG AGT -3'	30%
Gam42a	γ-Proteobacteria	5'- GCC TTC CCA CAT CGT TT -3'	30%
LGC354a-c	Firmicutes	a) 5'- TGG AAG ATT CCC TAC TGC -3'<br>b) 5'- CGG AAG ATT CCC TAC TGC -3'<br>c) 5'- CCG AAG ATT CCC TAC TGC -3'	30%
BAC303	Bacteroidetes	5'- CCA ATG TGG GGG ACC TT -3'	0%
